# Harnessing Gut Endocrine Cell Plasticity to Restore Insulin Production

**DOI:** 10.3390/cells15060544

**Published:** 2026-03-19

**Authors:** Chaïma Ayachi, Tiziana Napolitano, Serena Silvano, Sophie Giorgetti-Peraldi, Ahmed Mansouri, Raphaël Rapetti-Mauss, Hugo Fofo, Valentin Lepage, Laura Etasse, Caroline Treins, Loan Tran, Patrick Collombat

**Affiliations:** 1 Faculté des Sciences, Université Côte d’Azur, 06100 Nice, France; chaima.ayachi@univ-cotedazur.fr (C.A.); tiziana.napolitano@univ-cotedazur.fr (T.N.); serena.silvano@univ-cotedazur.fr (S.S.); sophie.giorgetti-peraldi@univ-cotedazur.fr (S.G.-P.); raphael.rapetti-mauss@univ-cotedazur.fr (R.R.-M.); hugo.fofo@univ-cotedazur.fr (H.F.); valentin.lepage@univ-cotedazur.fr (V.L.); laura.etasse@univ-cotedazur.fr (L.E.); caroline.treins@univ-cotedazur.fr (C.T.); loan.tran@univ-cotedazur.fr (L.T.); 2 iBV, Valrose Institute of Biology, Inserm, CNRS, Parc Valrose, 28 Avenue Valrose, 06108 Nice, France; 3 C3M, Mediterranean Center for Molecular Medicine, Inserm, 06103 Nice, France; 4 Department of Clinical Neurophysiology, University of Göttingen, 37075 Goettingen, Germany; amansou@mpinat.mpg.de

**Keywords:** gastrointestinal tract, L-cells, cellular reprogramming, *Pax4*, β-cell regeneration, type 1 diabetes

## Abstract

Type 1 diabetes (T1D) results from autoimmune-mediated destruction of pancreatic β-cells, leading to insulin deficiency and chronic hyperglycemia. β-cell replacement represents a promising therapeutic strategy, yet the identification of a sustainable and immune-compatible cell source remains a major challenge. Here, we explore the potential of the gastrointestinal (GI) epithelium as an alternative source of β-cells through in vivo cellular reprogramming. Given the large size and highly regenerative nature of the GI tract, partial reprogramming could provide a renewable source of insulin-producing (insulin^+^) cells. We demonstrate that ectopic expression of *Pax4* is sufficient to convert gut endocrine L-cells into insulin^+^ cells in vivo. Phenotypic analyses reveal that these gut-derived cells express key β-cell markers, components of the glucose-sensing machinery, and properly process proinsulin into mature insulin. Functional studies using organoids derived from *Pax4*-expressing gut epithelium further demonstrate that these cells display glucose-responsive insulin secretion. Collectively, our findings highlight the plasticity of gut endocrine cells and support the feasibility of generating β-like cells from the GI epithelium, providing a potential avenue for the development of alternative cell-based therapies for T1D.

## 1. Introduction

The autoimmune-mediated loss of β-cell mass in type 1 diabetes (T1D) has prompted intense efforts to identify alternative sources of insulin-producing (insulin^+^) cells capable of restoring endogenous insulin secretion and normoglycemia. Among these approaches, β-cell replacement has emerged as a promising strategy, either through differentiation of embryonic stem cells or direct reprogramming of non-β cells [1,2,3,4]. However, the autoimmune nature of T1D represents a major challenge for β-cell replacement strategies, as newly generated cells may remain susceptible to immune-mediated destruction.

Reprogramming endogenous endocrine cells into glucose-responsive β-cells represents an attractive avenue for developing autologous regenerative therapies in situ. However, newly generated β-cells may remain vulnerable to autoimmune recurrence. In this context, identifying a renewable, accessible, and potentially immune-compatible source of insulin^+^ cells is of particular interest.

The gastrointestinal (GI) epithelium constitutes a highly regenerative tissue containing abundant stem and endocrine cell populations [3,5]. Given its extensive surface area and rapid epithelial turnover, reprogramming even a limited fraction of gut epithelial cells could theoretically provide a sustainable source of β-like cells without compromising gut function [6]. Previous studies have established the feasibility of generating gut-derived β-like cells through Foxo1 inhibition or overexpression of pancreatic transcription factors (*Pdx1*, *MafA*, and *Ngn3*; PMN factors) [5,7,8,9,10]. Despite these promising approaches, limitations such as low conversion efficiency and potential adverse effects underscore the need for alternative and potentially simpler reprogramming strategies [3,11].

Paired Box 4 (Pax4) is a transcription factor critical for pancreatic β-cell and gut endocrine cell development, promoting lineage specification [12,13,14]. Beyond its developmental role, Pax4 has garnered significant attention for its potency as a reprogramming factor for generating insulin^+^ cells from non-β sources [12,15,16,17,18]. Notably, ectopic expression of *Pax4* is sufficient to convert pancreatic glucagon-producing (glucagon^+^) α-cells into functional β-like cells capable of ameliorating toxin-induced diabetes in vivo [18]. Interestingly, gut endocrine glucagon^+^ L-cells share key molecular and developmental features with pancreatic α-cells, including Ngn3-dependent differentiation as well as glucose-sensing and hormone secretion machinery [19,20,21,22].

Based on this rationale, we investigated whether ectopic *Pax4* expression could also induce the formation of insulin^+^ cells in the gut in vivo. Our findings demonstrate that *Pax4* alone is sufficient to generate gut-derived β-like cells expressing key β-cell markers and glucose sensing components. These cells are functionally capable of responding to glucose, supporting the concept that the GI epithelium may serve as a renewable source of self-sustaining β-like cells with potential applications for glycemic control.

## 2. Materials and Methods

### 2.1. Animal Manipulations

To induce targeted *Pax4* ectopic expression in glucagon^+^ L-cells, Gcg-Cre^ERT2^::Pax4-OE mice were generated by crossing Gcg-Cre^ERT2^ mice [23], expressing a tamoxifen (TAM)-inducible *Cre^ERT2^* recombinase knocked into the endogenous pre-proglucagon gene, with the previously described Pax4-OE mouse line [17].

For validation of Cre^ERT2^ recombination efficiency and specificity, Gcg-Cre^ERT2^ mice were also crossed with the Rosa26-β-gal reporter line [24], which carries a *loxP*-flanked transcriptional *STOP* cassette, including a neomycin resistance gene (NEO^R^), upstream of the β-*galactosidase* cDNA under the control of the ubiquitous *Rosa26 promoter*.

Animals were genotyped via GFP fluorescence and PCR for *Cre^ERT2^* and *β-galactosidase* genes. Recombination was induced in 8-week-old male mice by intraperitoneal injection of TAM (20 mg/mL; Sigma–Aldrich, St. Louis, MO, USA) daily for five consecutive days, combined with oral administration in drinking water (50 mg/L; Biogaran, Colombes, France), freshly prepared weekly.

### 2.2. Isolation of Murine Pancreatic Islets of Langerhans

Murine pancreatic islets were isolated from 8-week-old TAM-treated Gcg-Cre^ERT2^::Pax4-OE mice and control littermates by perfusing a collagenase solution (1 mg/mL; Sigma–Aldrich) diluted in Dulbecco’s Modified Eagle’s Medium (DMEM; Gibco, Thermo Fisher Scientific, Waltham, MA, USA) into the main pancreatic duct. Perfused pancreata were incubated at 37 °C for 12 min to allow enzymatic digestion, and the reaction was subsequently stopped by adding high-glucose DMEM containing 10% fetal bovine serum (FBS; Gibco). Following several centrifugation steps, islets were purified by density gradient separation using Histopaque (10771 and 11191; Merck, Darmstadt, Germany). Purified islets were then cultured in RPMI-1640 medium (Merck) supplemented with 10% FBS and 1% penicillin/streptomycin (10,000 U/mL; Gibco) in a humidified incubator at 37 °C with 5% CO_2_. After an overnight recovery period, islets were hand-picked under a stereomicroscope, and 300 islets per sample were collected into clean Eppendorf tubes for subsequent RNA extraction.

### 2.3. Immunohistochemistry

GI and pancreatic tissues were fixed in 4% paraformaldehyde (Microm Microtech, Francheville, France) for 30 min at 4 °C, embedded in paraffin, and sectioned at 5 μm. Sections were processed as previously described [25], with DAPI counterstaining.

Primary antibodies included: guinea pig anti-insulin (1:500; Dako, Glostrup, Denmark), mouse anti-glucagon (1:500; Sigma–Aldrich), rabbit anti-PC1/3 (1:500; Millipore, Burlington, MA, USA), anti-chromogranin A (CgA, 1:500; Abcam, Cambridge, UK), anti-GLUT-2 (1:500; Millipore), anti-glucose-dependent insulinotropic polypeptide (GIP, 1:100; Abcam), anti-Kir6.2 (1:500; Abcam), rat anti-C-peptide (1:500; Phoenix Pharmaceuticals, Burlingame, CA, USA), and chicken anti-β-galactosidase (1:500; Abcam). Secondary antibodies were Alexa Fluor 488, 594, and 647 conjugates (1:1000; Invitrogen, Thermo Fisher Scientific, Waltham, MA, USA).

Images were acquired using an Axioimager Z1 (Zeiss, Oberkochen, Germany) with appropriate filter sets and processed with Axiovision Rel. 4.8. For quantification of insulin^+^, glucagon^+^, and GIP^+^ cells, 100 CgA^+^ cells per animal were analyzed across at least 10 randomly selected sections from five animals per genotype.

Colonoids were stained following a modified protocol [26]. Organoid cultures were seeded in 4-well Nunc Lab-Tek chamber slides (Thermo Fisher Scientific, Waltham, MA, USA). Primary and secondary antibodies were used at 1:200 and 1:250, respectively. Images were captured using a Nikon Confocal Spinning Disk TiE W1 (Nikon, Tokyo, Japan) and processed with ImageJ 1.52n (National Institutes of Health, Bethesda, MD, USA).

### 2.4. Gene Expression Analysis

Total RNA was extracted from GI segments (fundus, corpus, antrum, duodenum, jejunum, ileum, and colon) using the RNeasy Mini Kit (Qiagen, Hilden, Germany), and from isolated pancreatic islets and colonoids using the RNeasy Micro Kit (Qiagen), according to the manufacturer’s instructions. RNA quantity and quality were assessed using a NanoDrop One spectrophotometer (Thermo Fisher Scientific).

Reverse transcription was performed with Superscript (Invitrogen), and quantitative PCR (qPCR) analyses were carried out using the QuantiTect SYBR Green Kit (Thermo Fisher Scientific) with validated mouse-specific primers (Qiagen). Gene expression levels were normalized to GAPDH and are reported as relative mRNA levels (fold-change).

### 2.5. Glucose Tolerance Tests and Blood Glucose Measurement

Mice were fasted for 6 h, then administered glucose (2 g/kg of body weight; Sigma-Aldrich) either orally (oGTT) or intraperitoneally (ipGTT). Blood glucose was measured at indicated intervals using an ONETOUCH Verio glucometer (LifeScan, Inc., Malvern, PA, USA).

### 2.6. Biochemical Analyses

Blood samples were collected at baseline, 10, and 15 min post-glucose administration. Plasma was isolated by cold centrifugation and stored at −80 °C. Plasma GLP-1 and GIP levels were measured using ELISA kits (CC-81508 and CC-81527; Crystal Chem, Itasca, IL, USA) following the manufacturer’s instructions.

### 2.7. Colonoid Generation, Maintenance, and 4-Hydroxytamoxifen Treatment

Colonoids were generated from colon crypts isolated from 8-week-old Gcg-Cre^ERT2^::Pax4-OE mice as previously described [10,27], with minor modifications. Crypts were embedded in Matrigel (Corning, Corning, NY, USA) and cultured in IntestiCult™ Organoid Growth Medium (STEMCELL Technologies, Vancouver, BC, Canada) supplemented with primocin (100 μg/mL; InvivoGen, San Diego, CA, USA) and penicillin/streptomycin (Gibco).

Colonoids were maintained at 37 °C, 5% CO_2_, and split 1:2 every 10–14 days. Two days after seeding, 10 μM 4-hydroxytamoxifen (4-OHT; Sigma–Aldrich) was applied for 72 h to induce recombination. Medium was then replaced with 4-OHT–free medium, and colonoids were harvested four days later for analysis.

### 2.8. Glucose-Stimulated Insulin Secretion (GSIS) Assay

Twenty to thirty colonoids were incubated in Krebs-Ringer buffer containing 2.8 mM glucose, 16.7 mM glucose, or 35 mM KCl. Secreted insulin was quantified using Mouse Ultrasensitive ELISA Kit (10-1243-01; Mercodia, Uppsala, Sweden) according to the manufacturer’s instructions.

### 2.9. Data Analysis

Data are presented as mean ± SEM from at least three independent animals. Normality was assessed with the D’Agostino–Pearson omnibus test. Statistical analyses were performed using GraphPad Prism 10 (GraphPad Software, San Diego, CA, USA). Differences were considered statistically significant at *p* < 0.05 (*), *p* < 0.01 (**), *p* < 0.001 (***), and *p* < 0.0001 (****).

## 3. Results

### 3.1. Generation and Characterization of Gcg-Cre^ERT2^::Pax4-OE Animals

**Figure 1:**
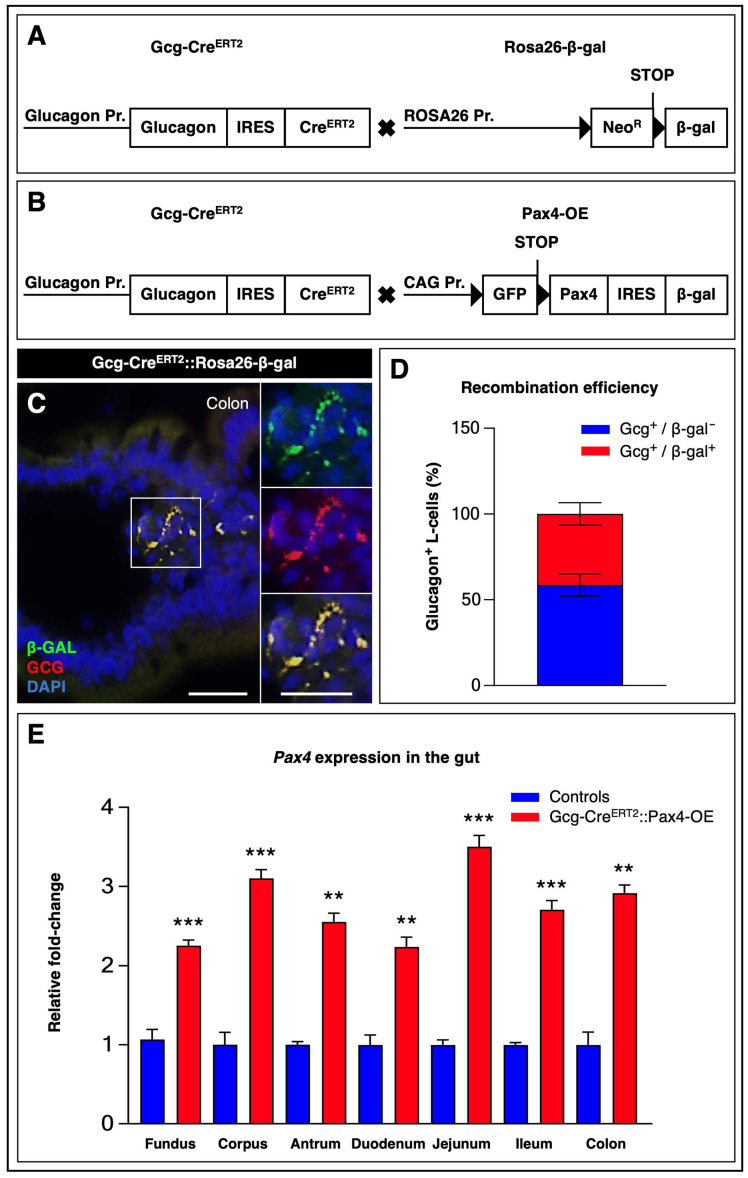
Generation and characterization of mice enabling *Pax4* ectopic expression in gut glucagon^+^ L-cells. (**A**) Control Gcg-Cre^ERT2^::Rosa26-gal mice were generated by crossing Gcg-Cre^ERT2^ animals [23], which express a TAM-inducible form of the *Cre^ERT2^* recombinase knocked into the endogenous *pre-proglucagon* locus, with the Rosa26-β-gal reporter line [24]. This reporter line carries a transgene consisting of the ubiquitous *Rosa26 promoter* upstream of a *loxP*-flanked transcriptional *STOP* cassette (including the NEO^R^ gene), followed by the *β-galactosidase* cDNA; (**B**) Gcg-Cre^ERT2^ mice were crossed with Pax4-OE animals [17], harboring a transgene composed of the ubiquitous *CAG promoter* driving a *loxP*-flanked *GFP-STOP* cassette followed by the *Pax4* cDNA linked to an IRES-β-galactosidase reporter. To induce recombination, double-transgenic male mice (8 weeks old) received TAM via daily intraperitoneal injections (20 mg/mL) for five consecutive days, combined with oral administration in drinking water (50 mg/L); (**C**) Immunohistochemical analysis of colonic paraffin sections from TAM-treated Gcg-Cre^ERT2^::Rosa26-β-gal mice. Representative images show co-detection of β-galactosidase (green) and glucagon (red), with yellow indicating colocalization. Nuclei were counterstained with DAPI (blue). Scale bar: 25 μm; (**D**) Quantification of the percentage of glucagon^+^ cells co-expressing β-galactosidase in the colon. A total of 100 glucagon^+^ cells were counted per mouse (*n* = 5); (**E**) *Pax4* mRNA levels were assessed by RT-qPCR in GI tissues. Relative mRNA expression levels were normalized to GAPDH and expressed as fold-change compared with control littermates (*n* = 3–5). Statistical significance was determined using an unpaired *t*-test with Welch’s correction (** *p* < 0.01, *** *p* < 0.001). Data are presented as mean ± SEM. Abbreviations: β-GAL, β-galactosidase; GCG, glucagon; GI, gastrointestinal; NEO^R^, neomycin resistance; PR, promoter; TAM, tamoxifen. See Figure S1.

To determine whether *Pax4* ectopic expression in gut glucagon^+^ L-cells could affect cell phenotype and identity in vivo, we first validated the specificity and efficiency of the Gcg-Cre^ERT2^ system. Gcg-Cre^ERT2^ mice [23], harboring a TAM-inducible *Cre^ERT2^* recombinase knocked into the endogenous *pre-proglucagon* locus, were crossed with the Rosa26-β-gal reporter line [24], which carries a *loxP*-flanked transcriptional *STOP* cassette—including the *neomycin resistance* gene—upstream of the *β-galactosidase* cDNA under the control of the ubiquitous *Rosa26 promoter* (Figure 1A).

Recombination efficiency was assessed in the colon, the GI segment displaying the highest density of L-cells [28]. Immunohistochemical analyses of colonic sections from TAM-treated Gcg-Cre^ERT2^::Rosa26-β-gal mice confirmed *Cre^ERT2^* activity exclusively in glucagon^+^ L-cells (Figure 1C). Quantitative analyses revealed recombination at the *Rosa26* locus in 41 ± 6.5% of L-cells (Figure 1D). Importantly, no glucagon^−^/β-galactosidase^+^ cells were detected, further confirming the specificity of *Cre^ERT2^*-mediated recombination to the L-cell lineage (Figure 1C).

Having validated the recombination efficiency and specificity of the model, Gcg-Cre^ERT2^ mice were subsequently crossed with Pax4-OE animals [17]. In this line, the transgene consists of the ubiquitous *CAG promoter* driving a *loxP-flanked GFP-STOP* cassette followed by the *Pax4* cDNA linked to an IRES-galactosidase reporter (Figure 1B). Because no reliable antibodies are currently available to specifically detect Pax4, its expression in GI tissues was assessed by RT-qPCR. Following TAM administration, *Pax4* mRNA expression was significantly increased in Gcg-Cre^ERT2^::Pax4-OE mice compared to control littermates, reaching up to approximately 3.5-fold induction depending on the GI region analyzed (Figure 1E; Figure S1A).

Importantly, Gcg-Cre^ERT2^::Pax4-OE mice were viable and fertile, with no evidence of premature mortality or overt health abnormalities.

### 3.2. Pax4 Misexpression in L-Cells Promotes Their Loss in the Gut

L-cells are classically distributed along the GI tract, from the upper intestine to the colon, with their density increasing towards the distal segments [28]. Using the Swiss-roll method to enable comprehensive scanning of the entire GI tract [29], we evaluated the impact of ectopic *Pax4* expression on L-cell populations and distribution.

**Figure 2:**
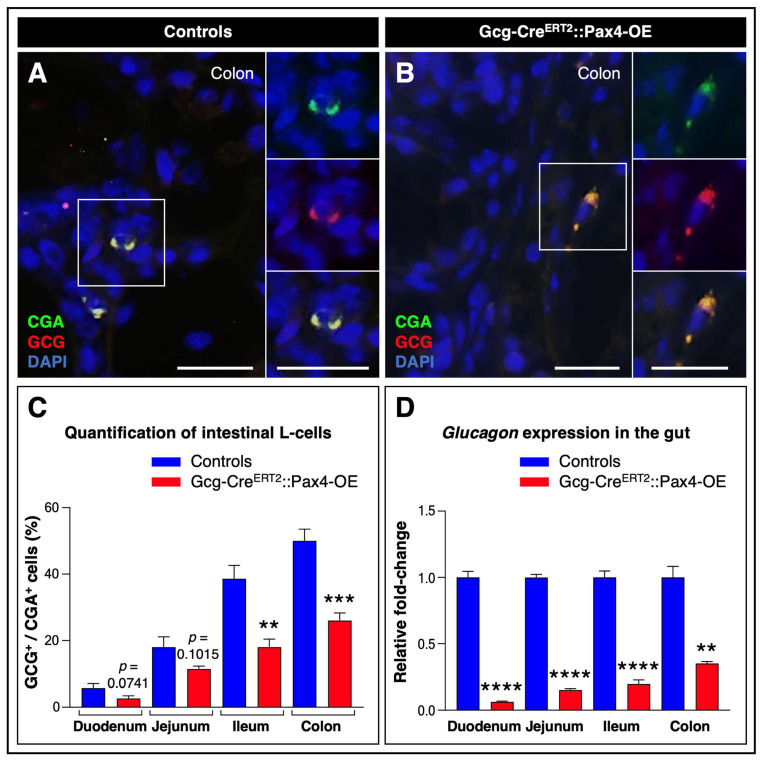
*Pax4* ectopic expression in L-cells promotes their depletion in the GI tract. (**A**,**B**) Immunohistochemical analysis of colonic paraffin sections from TAM-treated Gcg-Cre^ERT2^::Pax4-OE animals and age- and sex-matched controls. Representative images show co-detection of CgA, a marker of mature gut endocrine cells (green), and glucagon (red), with yellow indicating colocalization. Nuclei were counterstained with DAPI (blue). Scale bar: 20 μm; (**C**) Quantification of intestinal L-cells. A total of 100 CgA^+^ endocrine cells were counted per mouse (*n* = 5); (**D**) *Glucagon* mRNA levels were assessed by RT-qPCR. Relative mRNA expression levels were normalized to GAPDH and expressed as fold-change relative to control animals (*n* = 3–6). Statistical significance was determined using an unpaired *t*-test with Welch’s correction (** *p* < 0.01, *** *p* < 0.001, **** *p* < 0.0001). Data are presented as mean ± SEM. Abbreviations: CgA, chromogranin A; GCG, glucagon; GI, gastrointestinal; TAM, tamoxifen. See Figure S2.

Immunohistochemical analyses confirmed the presence of L-cells throughout the tract in both control and TAM-treated Gcg-Cre^ERT2^::Pax4-OE animals (Figure 2A,B).

Consistent with previous reports, quantitative analyses of glucagon^+^/chromogranin A^+^ cells (CgA, a common marker of mature gut endocrine cells) revealed higher L-cell counts in the ileum and colon compared with other intestinal segments [19] (Figure 2C).

Importantly, ectopic *Pax4* expression was associated with a significant reduction in L-cell numbers, with up to a 48 ± 8.2% decrease observed in the colon. This reduction was further supported by gene expression analyses, which showed a marked reduction in *glucagon* mRNA levels in transgenic tissues relative to controls (Figure 2D). Notably, *Pax4* misexpression did not significantly alter the expression of other gut hormones (Figure S2).

Together, these results indicate that *Pax4* misexpression in L-cells promotes their substantial loss throughout the GI tract.

### 3.3. Pax4 Ectopic Expression Drives the Conversion of Gut L-Cells into Insulin^+^ Cells

To determine whether the decrease in glucagon expression observed in *Pax4*-misexpressing gut tissues was accompanied by the acquisition of a β-like cell phenotype, as previously reported in the pancreas [17,18], additional quantitative immunohistochemical analyses were performed.

**Figure 3:**
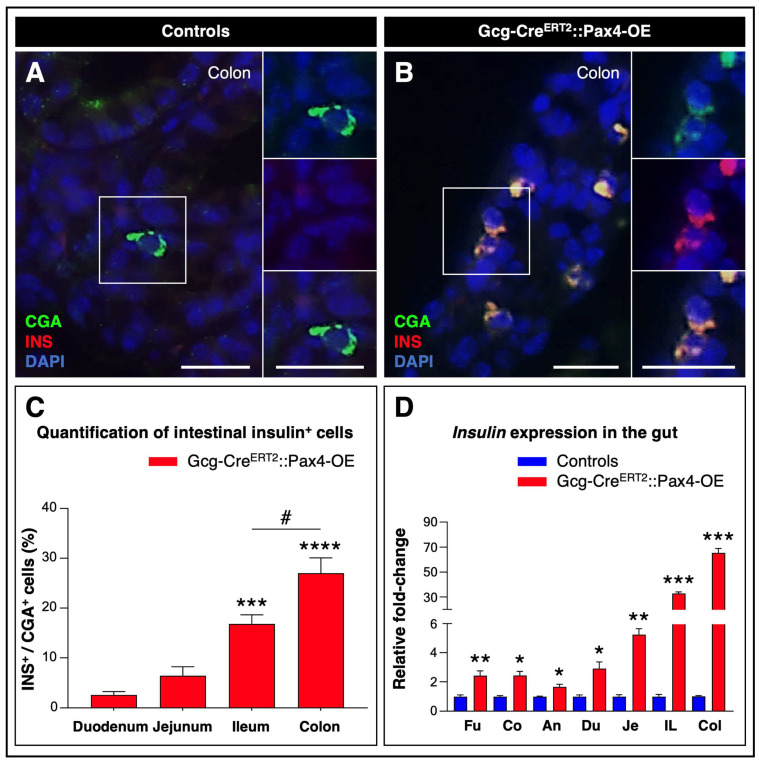
*Pax4* efficiently reprograms gut L-cells into insulin^+^ cells. (**A**,**B**) Immunohistochemical analysis of colonic paraffin sections from TAM-treated Gcg-Cre^ERT2^::Pax4-OE animals and age- and sex-matched controls. Representative images show co-detection of CgA (green) and insulin (red), with yellow indicating colocalization. Nuclei were counterstained with DAPI (blue). Scale bar: 20 μm; (**C**) Quantification of intestinal insulin^+^ cells. A total of 100 CgA^+^ endocrine cells were counted per mouse (*n* = 5); (**D**) *Insulin* mRNA levels were assessed by RT-qPCR. Relative mRNA expression levels were normalized to GAPDH and expressed as fold-change relative to control animals (*n* = 3–6). Statistical significance for quantification and gene expression analysis was determined using a one-way ANOVA with Bonferroni’s correction and an unpaired *t*-test with Welch’s correction, respectively (# *p* < 0.05, * *p* < 0.05, ** *p* < 0.01, *** *p* < 0.001, **** *p* < 0.0001). Data are presented as mean ± SEM. Abbreviations: AN, antrum; CO, colon; CR, corpus; CgA, chromogranin A; DU, duodenum; FU, fundus; IL, ileum; INS, insulin; JE, jejunum; TAM, tamoxifen. See Figures S3 and S4.

As expected, no insulin^+^ cells were detected along the GI tract in control animals (Figure 3A). In contrast, following TAM treatment of Gcg-Cre^ERT2^::Pax4-OE animals, numerous insulin^+^ cells were observed throughout the entire GI tract, with their numbers progressively increasing from the fundus to the colon (Figure 3B,C and Figure S3A–F).

In agreement with these observations, RT-qPCR analysis revealed a substantial increase in *insulin* mRNA levels in transgenic tissues compared to controls, reaching up to a 65.35 ± 3.55-fold induction in the colon (Figure 3D).

Among Gcg-Cre^ERT2^::Pax4-OE GI tissues, the colon appeared to represent the largest reservoir of insulin^+^ cells (Figure 3C,D). Accordingly, subsequent analyses focused on this region. Interestingly, a small subset of bihormonal cells co-expressing glucagon and insulin was detected exclusively in transgenic tissues (Figure S4A,B), suggesting the existence of a transitional state during the conversion of L-cells into insulin^+^ cells. Importantly, no co-expression of insulin with other gut hormones was observed.

To further investigate the fate of *Pax4*-misexpressing L-cells, β-galactosidase lineage tracing was performed by comparing TAM-treated Gcg-Cre^ERT2^::Rosa26-β-gal control tissues with TAM-treated Gcg-Cre^ERT2^::Pax4-OE tissues. In Gcg-Cre^ERT2^::Rosa26-β-gal sections, β-galactosidase^+^ cells were, as expected, scattered throughout the epithelium (Figure S4C), and none co-expressed insulin.

In contrast, in TAM-treated Gcg-Cre^ERT2^::Pax4-OE sections, approximately 20.6% of β-galactosidase^+^ cells expressed insulin (Figure S4D,E), in agreement with previous quantifications (Figure 3C). Notably, all insulin^+^ cells were consistently labeled with the β-galactosidase tracer, confirming their origin from glucagon-expressing L-cells. Although overall recombination efficiency reached approximately 41 ± 6.5% (Figure 1D), insulin expression was detected in a smaller proportion of lineage-traced cells in such 5-day-long analysis, consistent with a progressive acquisition of insulin expression following *Pax4* ectopic expression.

Collectively, these data demonstrate that *Pax4* misexpression alone induces the conversion of gut L-cells into insulin^+^ cells.

### 3.4. Gut-Derived Insulin^+^ Cells Display a β-like Cell Phenotype

To further characterize the molecular identity of the newly generated insulin^+^ cells, we performed comprehensive immunohistochemical analyses to assess the expression of bona fide β-cell markers in colonic sections from TAM-treated Gcg-Cre^ERT2^::Pax4-OE animals.

**Figure 4:**
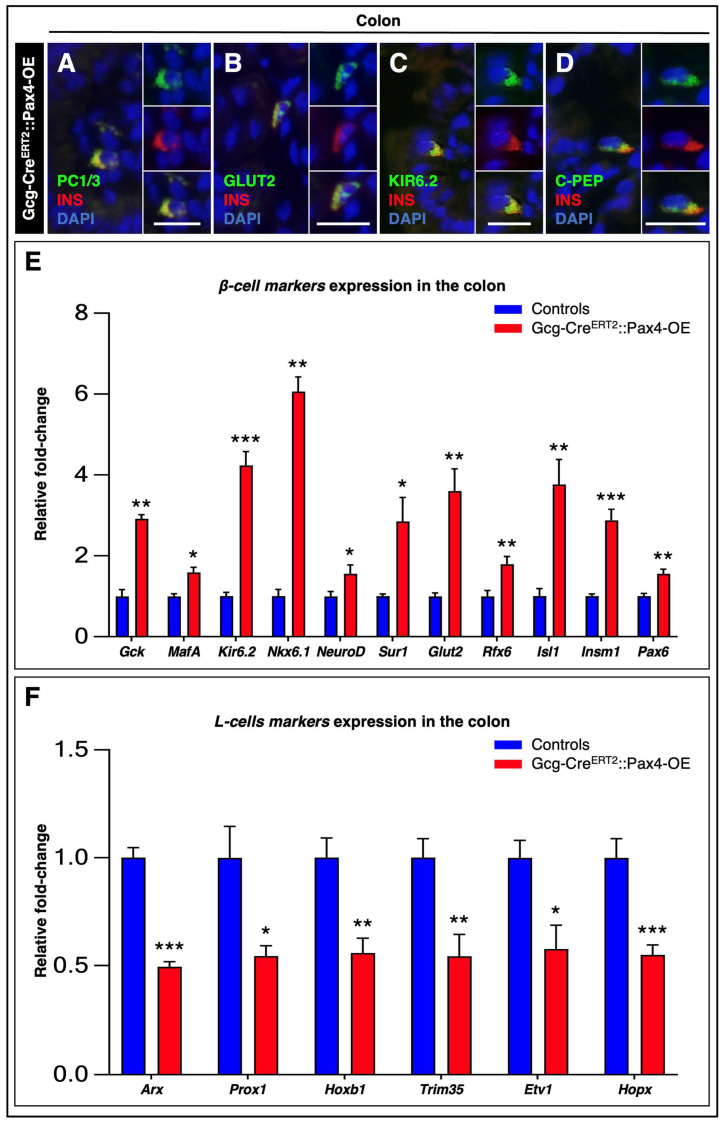
Gut-derived insulin^+^ cells share substantial phenotypic similarity with pancreatic β-cells. (**A**–**D**) Co-immunohistochemical analysis of insulin (red) together with PC1/3, GLUT2, Kir6.2, or C-peptide (green) on representative colonic paraffin sections from TAM-treated Gcg-Cre^ERT2^::Pax4-OE animals, with yellow indicating colocalization. Nuclei were counterstained with DAPI (blue). Scale bar: 20 μm; (**E**) β-cell- and (**F**) L-cell-associated markers mRNA levels were assessed by RT-qPCR. Relative mRNA expression levels were normalized to GAPDH and expressed as fold-change relative to control animals (*n* = 4–7). Statistical significance was determined using an unpaired *t*-test with Welch’s correction or a Mann–Whitney test (* *p* < 0.05, ** *p* < 0.01, *** *p* < 0.001). Data are presented as mean ± SEM. Abbreviations: C-PEP, C-peptide; INS, insulin; TAM, tamoxifen.

Our analyses revealed that all colonic insulin^+^ cells uniformly expressed key β-cell markers, including PC1/3 (required for insulin maturation; Figure 4A), GLUT2 (a glucose transporter involved in glucose sensing; Figure 4B), Kir6.2 (the principal pore-forming subunit for the K_ATP_ channel involved in glucose-stimulated insulin secretion (GSIS); Figure 4C), and C-peptide (a byproduct of insulin biosynthesis; Figure 4D).

To further substantiate these findings and overcome limitations related to antibody availability, we analyzed the expression of a broad panel of β-cell-associated genes by RT-qPCR. These analyses revealed a significant upregulation of β-cell-specific mRNA levels in transgenic tissues relative to controls (Figure 4E). Notably, critical regulators of β-cell development and function—including *Nkx6.1*, *NeuroD1*, *Isl1*, *Rfx6*, *Insm1*, *Sur1*, and *Gck*—were significantly enriched in Gcg-Cre^ERT2^::Pax4-OE tissues, supporting the acquisition of a β-like cell phenotype by gut-derived insulin^+^ cells.

Concomitantly, we observed a marked decrease in the expression of multiple L-cell-associated markers [30], including *Arx*, *Prox1*, *Hoxb1*, *Trim35*, *Etv1*, and *Hopx*, in *Pax4*-misexpressing tissues compared with controls (Figure 4F).

Collectively, these results indicate that *Pax4*-misexpressing L-cells progressively lose their original identity while acquiring a β-like cell phenotype.

### 3.5. Improved Glucose Tolerance in Pax4-Misexpressing Mice Is Associated with Functional Gut Insulin^+^ Cells and Compensatory K-Cell Expansion

To determine whether gut-derived insulin^+^ cells were functionally competent and capable of improving glucose handling, we performed an ipGTT on TAM-treated Gcg-Cre^ERT2^::Pax4-OE animals and matched controls.

**Figure 5:**
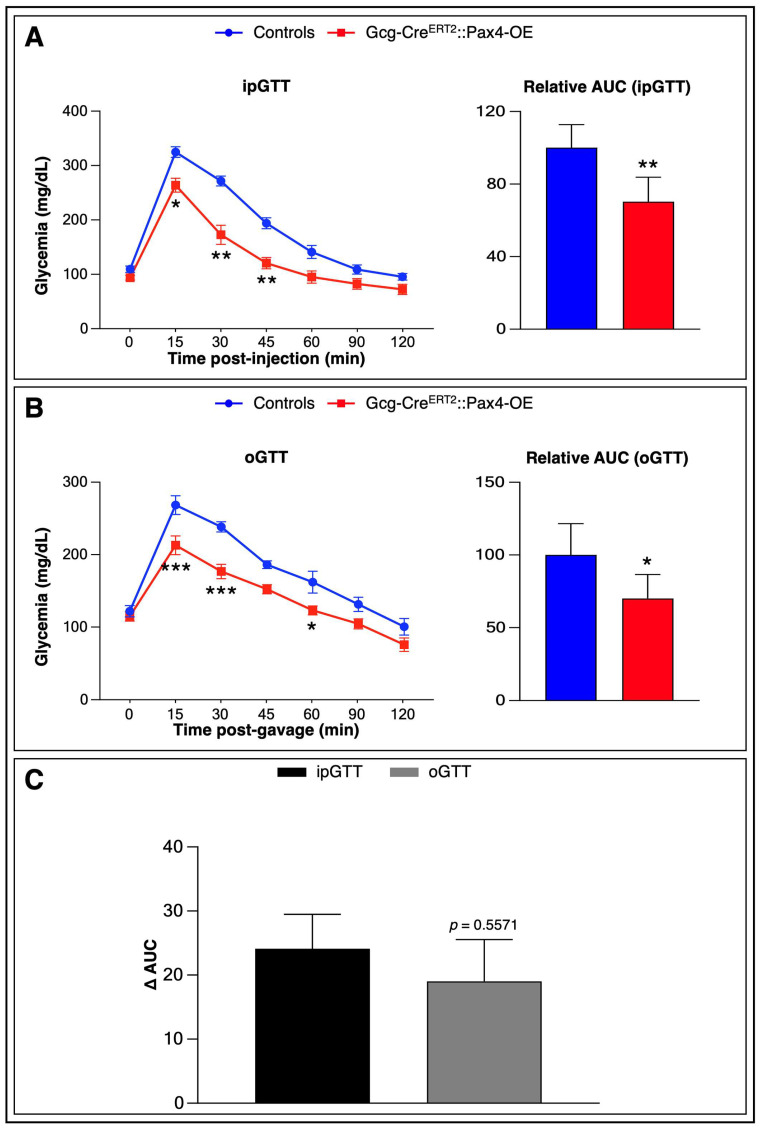
Gut-derived insulin^+^ cells improve glucose tolerance in *Pax4*-misexpressing animals. (**A**) ipGTT (2 g/kg) and corresponding AUC analysis in control and TAM-treated Gcg-Cre^ERT2^::Pax4-OE animals (*n* = 7). Relative AUC values are expressed as a percentage of the mean AUC of control animals; (**B**) oGTT (2 g/kg) and AUC analysis in the same animals; (**C**) Comparative analysis of AUCs between ipGTT and oGTT (ΔAUC). For GTT and AUC analyses, statistical significance was determined using a two-way ANOVA with Bonferroni’s correction or an unpaired *t*-test with Welch’s correction, respectively (* *p* < 0.05, ** *p* < 0.01, *** *p* < 0.001). Data are presented as mean ± SEM. Abbreviations: AUC, area under the curve; ipGTT, intraperitoneal glucose tolerance test; oGTT, oral glucose tolerance test; TAM, tamoxifen. See Figure S5.

Analysis of glycemic curves revealed a significant improvement in glucose tolerance in transgenic mice, characterized by a reduced glycemic peak and a faster return to fasting glycemia compared to controls (Figure 5A), suggesting an increase in functional β-cell mass and/or enhanced insulin-secreting capacity.

Because L-cells play a critical role in glucose homeostasis through the secretion of glucagon-like peptide 1 (GLP-1), an intestinal hormone that enhances GSIS, we next examined whether their conversion into insulin^+^ cells affected GLP-1 production. Consistent with the reduction in L-cell numbers (Figure 2C), glucose-stimulated GLP-1 levels were significantly decreased in transgenic animals compared to controls (Figure S5A). However, this reduction had limited metabolic impact.

Indeed, during an oGTT, Gcg-Cre^ERT2^::Pax4-OE mice again displayed significantly improved glucose tolerance, with no substantial difference in ΔAUC compared to ipGTT results (Figure 5B,C). These findings suggest the involvement of compensatory mechanisms, potentially involving K-cells, which secrete the incretin hormone glucose-dependent insulinotropic polypeptide (GIP) and may help preserve glucose homeostasis.

**Figure 6:**
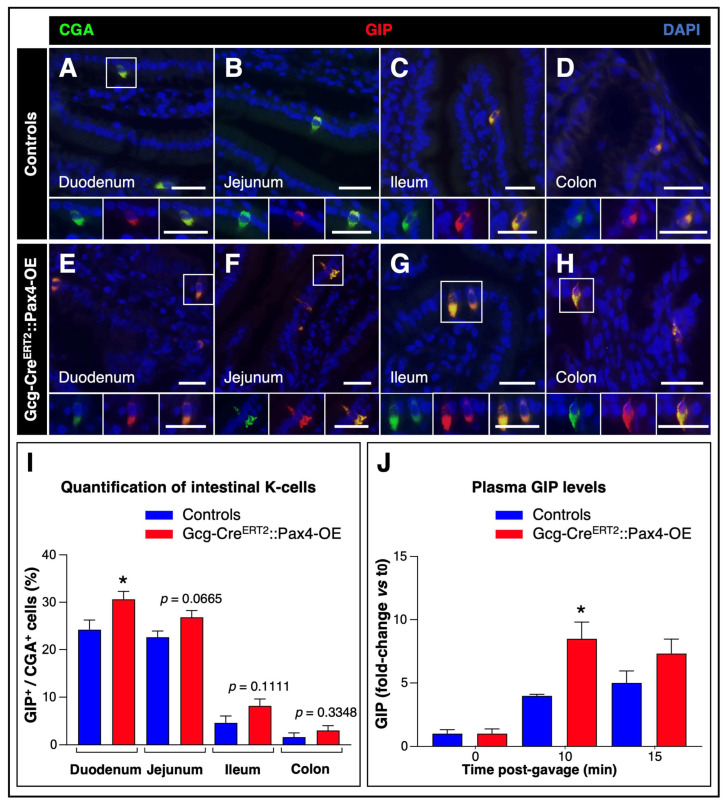
Compensatory mechanisms in *Pax4*-misexpressing animals. (**A**–**H**) Immunohistochemical analysis of intestinal paraffin sections from TAM-treated Gcg-Cre^ERT2^::Pax4-OE animals and age- and sex-matched controls. Representative images show co-detection of CgA (green) and GIP (red), with yellow indicating colocalization. Nuclei were counterstained with DAPI (blue). White boxes highlight representative cells of interest that are enlarged below each panel. Scale bar: 25 μm; (**I**) Quantification of intestinal K-cells. A total of 100 CgA^+^ endocrine cells were counted per mouse (*n* = 5); (**J**) Plasma GIP levels were measured at baseline, and 10 and 15 min after oral glucose administration (2 g/kg) in control and TAM-treated Gcg-Cre^ERT2^::Pax4-OE mice (*n* = 6–7). Values are normalized to t = 0 min for each genotype. Statistical significance was determined using an unpaired *t*-test with Welch’s correction or a Mann–Whitney test (* *p* < 0.05). Data are presented as mean ± SEM. Abbreviations: CgA, chromogranin A; GIP, glucose-dependent insulinotropic polypeptide; TAM, tamoxifen.

To investigate this possibility, we first examined the distribution of GIP^+^ K-cells and confirmed their presence in both control and transgenic tissues (Figure 6A–H).

Notably, we observed a marked increase in K-cell numbers in Gcg-Cre^ERT2^::Pax4-OE intestines, reaching up to approximately 87.5% depending on the intestinal segment analyzed (Figure 6I).

Consistent with this observation, plasma GIP levels following the oral glucose challenge were increased in transgenic mice compared to controls, reaching statistical significance at 10 min and showing a trend toward significance at 15 min (*p* = 0.1431) (Figure 6J). Together, these results indicate that compensatory expansion of K-cells occurs in transgenic animals, likely counterbalancing L-cell loss and contributing to preserved incretin responsiveness.

Importantly, the improved glucose tolerance observed in Gcg-Cre^ERT2^::Pax4-OE mice cannot solely be attributed to gut-derived insulin^+^ cells and compensatory K-cells. This model also induces the conversion of pancreatic glucagon^+^ α-cells into insulin^+^ cells [17,18], which may additionally contribute to enhanced glucose control.

### 3.6. Bioengineered Pax4-Misexpressing Mini-Guts Release Insulin upon Glucose Stimulation

To circumvent the intrinsic limitations of the in vivo murine model and directly assess the functional competence of gut-derived insulin^+^ cells, we implemented an ex vivo approach using bioengineered murine mini-guts. Colon-derived organoids (colonoids) were generated from Gcg-Cre^ERT2^::Pax4-OE mice and corresponding controls.

**Figure 7:**
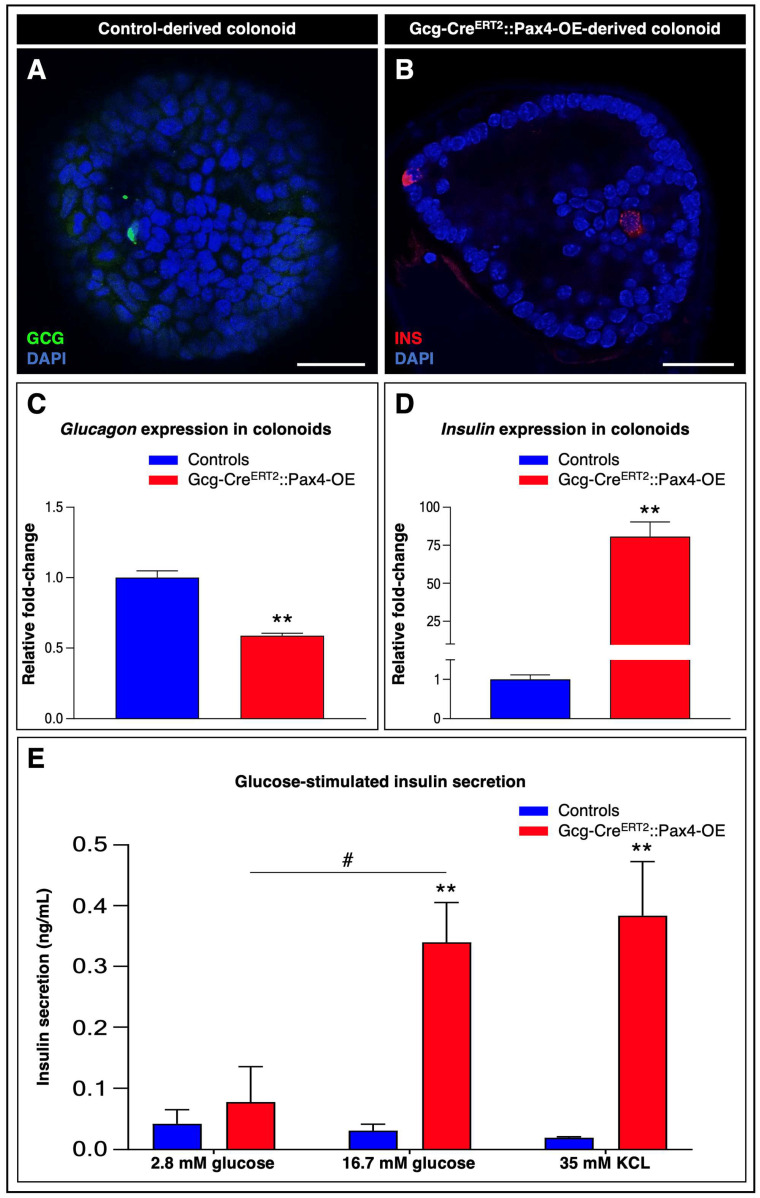
Gut-derived insulin^+^ cells generated ex vivo are functional and glucose-responsive. (**A**,**B**) Immunofluorescent detection of glucagon (green) in control colonoids (**A**) and insulin (red) in Gcg-Cre^ERT2^::Pax4-OE colonoids treated in vitro for 72 h with 10 µM 4-OHT (**B**). Nuclei were counterstained with DAPI (blue). Scale bar: 25 μm; (**C**,**D**) *Glucagon* and *insulin* mRNA levels were assessed by RT-qPCR. Relative mRNA expression levels were normalized to GAPDH and expressed as fold-change relative to controls (*n* = 4); (**E**) Insulin secretion from control and 4-OHT-treated Gcg-Cre^ERT2^::Pax4-OE colonoids was assessed under low glucose (2.8 mM), high glucose (16.7 mM), or 35 mM KCl. Secreted insulin was quantified by ELISA. Statistical significance was determined using an unpaired *t*-test with Welch’s correction or a Mann–Whitney test (# *p* < 0.05, ** *p* < 0.01). Data are presented as mean ± SEM. Abbreviations: 4-OHT, 4-hydroxytamoxifen; GCG, glucagon; INS, insulin. See Figure S6.

We first determined whether ex vivo *Pax4* misexpression in L-cells could recapitulate the phenotype observed in vivo. Immunohistochemical analyses confirmed the presence of glucagon^+^ L-cells (Figure 7A) and revealed insulin^+^ cells exclusively in 4-OHT-treated Gcg-Cre^ERT2^::Pax4-OE colonoids (Figure 7B).

Consistent with these observations, RT-qPCR analyses demonstrated a significant reduction in *glucagon* mRNA levels (~41.24%; Figure 7C) together with a marked increase in *insulin* transcripts (79.67 ± 9.67-fold; Figure 7D) in transgenic colonoids compared to controls.

Further characterization identified bihormonal (glucagon^+^/insulin^+^) cells exclusively in *Pax4*-misexpressing colonoids (Figure S6A), suggesting an intermediate conversion state. Lineage-tracing experiments confirmed that insulin^+^ cells originated from L-cells ex vivo (Figure S6B). Moreover, expression analyses of canonical β-cell markers demonstrated that these insulin^+^ cells exhibited a bona fide β-cell molecular signature (Figure S6C–E). Collectively, these findings validate *Pax4*-misexpressing colonoids as a *robust* ex vivo platform to investigate the functionality of gut-derived insulin^+^ cells.

To directly evaluate their secretory competence, we performed GSIS assays on colonoids derived from control and Gcg-Cre^ERT2^::Pax4-OE animals. Exposure to low glucose (2.8 mM) did not induce detectable insulin secretion in either group (Figure 7E). In contrast, high glucose stimulation (16.7 mM) induced robust insulin release exclusively in *Pax4*-misexpressing colonoids, corresponding to an approximately 4.36-fold increase compared to basal conditions. Additional validation was obtained using 35 mM KCl, a well-established depolarizing agent that induces insulin vesicle exocytosis [31,32], which similarly resulted in a significant increase in insulin release in transgenic colonoids (Figure 7E).

Together, these results demonstrate that gut-derived insulin^+^ cells generated through *Pax4* misexpression are not only capable of producing insulin but also exhibit glucose-regulated secretory activity characteristic of functional β-like cells.

## 4. Discussion

In this study, we demonstrate that gut endocrine cells represent a potential source for cellular reprogramming to replace lost pancreatic β-cells in T1D. Our data show that ectopic *Pax4* expression is sufficient to convert gut L-cells into insulin^+^ cells exhibiting key molecular and functional hallmarks of authentic β-cells. Using bioengineered *Pax4*-misexpressing mini-guts, we further confirmed their functionality ex vivo, providing compelling evidence that patient-specific gut-derived β-like cells may represent a promising strategy to restore normoglycemia in T1D.

### 4.1. Ectopic Pax4 Expression Converts Gut L-Cells into Insulin^+^ Cells

Previous studies demonstrated that ectopic *Pax4* expression in pancreatic glucagon^+^ α-cells is sufficient to convert them into β-like cells [17,18]. Given the abundance of glucagon^+^ L-cells in the gut and their molecular similarities to pancreatic α-cells, we investigated whether a comparable reprogramming event could be induced in the GI epithelium. Unlike earlier studies in which the minimal *glucagon promoter* restricted *Pax4* expression primarily to pancreatic α-cells [17,18], we used a Gcg-Cre^ERT2^ mouse line with *Cre^ERT2^* inserted into the endogenous *pre-proglucagon* locus [23], thereby enabling targeted *Pax4* misexpression in gut L-cells.

This strategy resulted in the generation of insulin^+^ cells throughout the GI tract, accompanied by a partial reduction in L-cell numbers. Importantly, the spatial distribution of newly generated insulin^+^ cells closely paralleled the endogenous distribution of L-cells, with the highest conversion efficiency observed in the distal intestine and colon. This observation is consistent with the higher density of L-cells reported in these regions [19].

Although the loss of gut endocrine cells has been associated with metabolic disturbances, including impaired lipid absorption and altered glucose homeostasis [33], *Pax4*-misexpressing animals displayed no overt physiological abnormalities. This likely reflects the remarkable regenerative capacity of the GI epithelium, which continuously replenishes endocrine populations through intestinal stem cell activity, as well as the partial *Cre^ERT2^* recombination efficiency observed in our model.

Following five consecutive days of TAM treatment, insulin^+^ cells were detected throughout the GI tract. Compared with shorter treatment regimens, this protocol proved to be the most efficient, as it encompassed a broader temporal window including both differentiating and mature L-cells susceptible to Pax4-mediated reprogramming. Notably, prolonged TAM exposure did not further increase the number of insulin^+^ cells, suggesting a cellular turnover rate comparable to that of native gut endocrine cells, estimated at approximately five to seven days [34,35]. Consistent with this interpretation, withdrawal of TAM resulted in the disappearance of intestinal insulin^+^ cells within one week, correlating with a partial decline in glycemic control (Figure S7A,B).

Lineage tracing analyses provided strong evidence that L-cells gradually transition toward a β-like identity. Specifically, L-cells initially adopt a transient glucagon^+^/insulin^+^ bihormonal phenotype before acquiring a more complete β-like transcriptional program. Such intermediate states have been observed in multiple endocrine reprogramming models and likely reflect the progressive remodeling of transcriptional networks required to stabilize the new cellular identity [17,18,36].

Importantly, only a subset of lineage-traced cells expressed insulin this 5-day-long analysis, reflecting the progressive nature of the conversion process. Further quantitative studies would therefore be required to determine the full efficiency of the reprogramming process and to reconcile the discrepancy between the proportion of recombined cells and the fraction of insulin^+^ cells detected in gut tissues.

Molecular characterization revealed that gut-derived insulin^+^ cells expressed key β-cell markers involved in insulin synthesis, processing, and secretion. In parallel, we observed a marked downregulation of several L-cell-associated genes, including *Arx*. This observation is consistent with the well-established antagonistic relationship between *Pax4* and *Arx* during endocrine lineage specification. As previously reported [14], *Arx* expression is largely confined to nascent hormone-producing cells rather than fully differentiated ones, supporting the hypothesis that Pax4-mediated reprogramming may primarily target early differentiating L-cells.

As an initial step toward elucidating the molecular mechanisms underlying this conversion, we examined the expression of *Cdx-2*, a transcription factor that acts as a master regulator of intestinal identity and has previously been shown to impede β-cell reprogramming [8,37]. Interestingly, *Cdx2* expression was markedly reduced in colonic tissues from *Pax4*-misexpressing animals. These findings raise the possibility that Pax4, or some of its downstream targets, may partially suppress intestinal lineage programs, thereby facilitating the acquisition of β-cell characteristics. Nevertheless, future genome-wide transcriptional and epigenomic analyses will be required to fully elucidate the regulatory networks governing Pax4-driven reprogramming in gut endocrine cells.

A limitation of the present study relates to the genetic model used to induce *Pax4* misexpression. As the Gcg-Cre^ERT2^ mouse line drives recombination in all glucagon-expressing cells [23], *Pax4* overexpression is not solely restricted to gut L-cells and may also occur in pancreatic α-cells. Additional analyses of pancreatic sections confirmed recombination in peripheral α-cells but did not reveal detectable α-to-β cell conversion during the short induction period used in this study (Figure S8A). Consistently, analysis of isolated pancreatic islets revealed a significant increase in *Pax4* mRNA levels without detectable changes in *insulin* expression (Figure S8B). One likely explanation is that the relatively brief TAM exposure may induce *Pax4* expression without allowing sufficient time for complete cellular conversion in the pancreas. In contrast, the rapid turnover and regenerative dynamics of the intestinal epithelium may facilitate faster cellular reprogramming events [38], potentially explaining the preferential detection of insulin^+^ cells in gut tissues.

### 4.2. Pax4-Misexpressing Animals Display Improved Glucose Metabolism

Functional analyses demonstrated that *Pax4*-misexpressing animals exhibited improved glucose tolerance. Despite a reduction in L-cell numbers and decreased glucose-stimulated GLP-1 secretion, transgenic mice maintained enhanced glycemic control. This observation indicates that newly generated insulin^+^ cells are functionally competent and may contribute to improved glucose handling.

Interestingly, the loss of L-cells was accompanied by a significant increase in K-cell counts and enhanced GIP secretion. These findings suggest the presence of compensatory mechanisms within the entero-insular axis. Similar adaptive responses have been reported in GLP-1 receptor knockout models, where increased GIP signaling contributes to maintaining glucose homeostasis [39,40]. Our observations therefore highlight the remarkable plasticity of intestinal endocrine populations and suggest that alterations in one enteroendocrine lineage may trigger compensatory expansion of others.

In addition, pancreatic GLP-1 and glucagon may function as local gluco-incretins capable of sustaining insulin secretion when intestinal GLP-1 levels are reduced [40,41,42]. Whether these mechanisms operate synergistically with gut-derived insulin^+^ cells to improve glycemic control remains an important question for future investigation.

### 4.3. Pax4-Misexpressing Mini-Guts Demonstrate Functional Insulin^+^ Cells

Given the potential physiological consequences of L-cell conversion within the intestinal epithelium [13,33,43], we developed bioengineered mini-guts as an ex vivo platform to study Pax4-mediated reprogramming. Organoid analyses confirmed that gut-derived insulin^+^ cells express canonical β-cell-specific markers and respond to glucose stimulation by secreting insulin.

These findings provide direct evidence that Pax4-mediated reprogramming generates functionally competent β-like cells capable of regulated insulin secretion. Importantly, the organoid system offers a powerful experimental platform to dissect the molecular mechanisms of gut endocrine reprogramming and to evaluate potential therapeutic applications.

Future transplantation experiments into chemically induced diabetic NOD-*scid* IL2Rg^null^ (NSG) mice will be essential to determine whether gut-derived β-like cells can restore normoglycemia in vivo [44,45].

Compared with previously described reprogramming approaches, including PMN factors overexpression or Foxo1 deletion [8,10], our strategy presents several advantages: (i) ectopic expression of a single factor, Pax4, is sufficient to induce a physiologically relevant β-cell phenotype; (ii) conversion occurs throughout the GI tract, providing a potentially abundant source of reprogrammable cells; and (iii) Foxo1 function remains intact, preserving its tumor-suppressive and β-cell-protective roles [11,46,47,48,49]. Taken together, these features suggest that Pax4-mediated reprogramming may represent a simpler and potentially safer strategy for generating β-like cells.

### 4.4. Conclusions and Perspectives

Our study demonstrates that *Pax4* misexpression enables the generation of insulin^+^ cells in the GI epithelium and highlights the remarkable plasticity of gut endocrine populations. These findings suggest that gut-derived β-like cells could represent a promising cellular source for β-cell replacement therapies.

Importantly, the gastrointestinal tract represents an accessible tissue that can be sampled through minimally invasive procedures such as endoscopic biopsies. This raises the possibility of generating patient-specific β-like cells using autologous gut tissue.

Future work would therefore be required to determine whether similar reprogramming events can be induced in human intestinal organoids and whether small molecules capable of mimicking Pax4 activity could be identified. Another critical question would be whether gut-derived β-like cells could evade autoimmune destruction in the context of T1D. Addressing these challenges would be essential to putatively translate Pax4-mediated reprogramming strategies into clinically relevant therapies.

## Data Availability

The datasets generated and/or analyzed during the current study are available from the corresponding author upon reasonable request. The data are not publicly available as no dedicated public repository is available for this type of dataset.

## Supplementary Materials

The following supporting information can be downloaded at: https://www.mdpi.com/article/10.3390/cells15060544/s1, Figure S1: *Pax4* expression is significantly increased throughout the GI tract of Gcg-Cre^ERT2^::Pax4-OE animals; Figure S2: *Pax4* misexpression did not significantly affect the expression of other gut hormones in the colon; Figure S3: *Pax4* misexpression in L-cells induces ectopic insulin^+^ cell formation throughout the GI tract; Figure S4: Lineage tracing analysis reveals the conversion of L-cells into insulin^+^ cells upon *Pax4* ectopic expression; Figure S5: Impaired GLP-1 secretion during oGTT in *Pax4*-misexpressing animals; Figure S6: Conversion of L-cells into β-like cells in *Pax4*-misexpressing colonoids; Figure S7. Pulse–chase analysis of glucose tolerance following tamoxifen washout and re-administration; Figure S8. *Pax4* ectopic expression in α-cells does not induce insulin^+^ cell formation in pancreatic islets.

## Abbreviations

The following abbreviations are used in this manuscript:

4-OHT	4-Hydroxytamoxifen
CGA	Chromogranin A
GFP	Green fluorescent protein
GI	Gastrointestinal
GIP	Glucose-dependent insulinotropic polypeptide
GLP-1	Glucagon-like peptide 1
GSIS	Glucose-stimulated insulin secretion
ipGTT	Intraperitoneal glucose tolerance test
NEO^R^	Neomycin resistance
oGTT	Oral glucose tolerance test
PAX4	Paired Box 4
PMN	Pdx1, MafA, and Ngn3
PYY	Peptide YY
SST	Somatostatin
TAM	Tamoxifen

## References

[1] Kalo E., Read S., Ahlenstiel G. (2022). Reprogramming—Evolving Path to Functional Surrogate beta-Cells. Cells.

[2] Wei R., Hong T. (2016). Lineage Reprogramming: A Promising Road for Pancreatic beta Cell Regeneration. Trends Endocrinol. Metab..

[3] McKimpson W.M., Accili D. (2019). Reprogramming Cells to Make Insulin. J. Endocr. Soc..

[4] Basile G., Qadir M.M.F., Mauvais-Jarvis F., Vetere A., Shoba V., Modell A.E., Pastori R.L., Russ H.A., Wagner B.K., Dominguez-Bendala J. (2022). Emerging diabetes therapies: Bringing back the beta-cells. Mol. Metab..

[5] Baafi K., March J.C. (2023). Harnessing gut cells for functional insulin production: Strategies and challenges. Biotechnol. Notes.

[6] Helander H.F., Fandriks L. (2014). Surface area of the digestive tract-revisited. Scand. J. Gastroenterol..

[7] Chen Y.J., Finkbeiner S.R., Weinblatt D., Emmett M.J., Tameire F., Yousefi M., Yang C., Maehr R., Zhou Q., Shemer R. (2014). De novo formation of insulin-producing “neo-beta cell islets” from intestinal crypts. Cell Rep..

[8] Ariyachet C., Tovaglieri A., Xiang G., Lu J., Shah M.S., Richmond C.A., Verbeke C., Melton D.A., Stanger B.Z., Mooney D. (2016). Reprogrammed Stomach Tissue as a Renewable Source of Functional beta Cells for Blood Glucose Regulation. Cell Stem Cell.

[9] Bouchi R., Foo K.S., Hua H., Tsuchiya K., Ohmura Y., Sandoval P.R., Ratner L.E., Egli D., Leibel R.L., Accili D. (2014). FOXO1 inhibition yields functional insulin-producing cells in human gut organoid cultures. Nat. Commun..

[10] Talchai C., Xuan S., Kitamura T., DePinho R.A., Accili D. (2012). Generation of functional insulin-producing cells in the gut by Foxo1 ablation. Nat. Genet..

[11] Yadav R.K., Chauhan A.S., Zhuang L., Gan B. (2018). FoxO transcription factors in cancer metabolism. Semin. Cancer Biol..

[12] Napolitano T., Avolio F., Courtney M., Vieira A., Druelle N., Ben-Othman N., Hadzic B., Navarro S., Collombat P. (2015). Pax4 acts as a key player in pancreas development and plasticity. Semin. Cell Dev. Biol..

[13] Schonhoff S.E., Giel-Moloney M., Leiter A.B. (2004). Minireview: Development and differentiation of gut endocrine cells. Endocrinology.

[14] Beucher A., Gjernes E., Collin C., Courtney M., Meunier A., Collombat P., Gradwohl G. (2012). The homeodomain-containing transcription factors Arx and Pax4 control enteroendocrine subtype specification in mice. PLoS ONE.

[15] Liew C.G., Shah N.N., Briston S.J., Shepherd R.M., Khoo C.P., Dunne M.J., Moore H.D., Cosgrove K.E., Andrews P.W. (2008). PAX4 enhances beta-cell differentiation of human embryonic stem cells. PLoS ONE.

[16] Druelle N., Vieira A., Shabro A., Courtney M., Mondin M., Rekima S., Napolitano T., Silvano S., Navarro-Sanz S., Hadzic B. (2017). Ectopic expression of Pax4 in pancreatic delta cells results in beta-like cell neogenesis. J. Cell Biol..

[17] Collombat P., Xu X., Ravassard P., Sosa-Pineda B., Dussaud S., Billestrup N., Madsen O.D., Serup P., Heimberg H., Mansouri A. (2009). The ectopic expression of Pax4 in the mouse pancreas converts progenitor cells into alpha and subsequently beta cells. Cell.

[18] Al-Hasani K., Pfeifer A., Courtney M., Ben-Othman N., Gjernes E., Vieira A., Druelle N., Avolio F., Ravassard P., Leuckx G. (2013). Adult duct-lining cells can reprogram into beta-like cells able to counter repeated cycles of toxin-induced diabetes. Dev. Cell.

[19] Kuhre R.E., Deacon C.F., Holst J.J., Petersen N. (2021). What Is an L-Cell and How Do We Study the Secretory Mechanisms of the L-Cell?. Front. Endocrinol..

[20] Reimann F., Habib A.M., Tolhurst G., Parker H.E., Rogers G.J., Gribble F.M. (2008). Glucose sensing in L cells: A primary cell study. Cell Metab..

[21] Jenny M., Uhl C., Roche C., Duluc I., Guillermin V., Guillemot F., Jensen J., Kedinger M., Gradwohl G. (2002). Neurogenin3 is differentially required for endocrine cell fate specification in the intestinal and gastric epithelium. EMBO J..

[22] Gehart H., van Es J.H., Hamer K., Beumer J., Kretzschmar K., Dekkers J.F., Rios A., Clevers H. (2019). Identification of Enteroendocrine Regulators by Real-Time Single-Cell Differentiation Mapping. Cell.

[23] Ackermann A.M., Zhang J., Heller A., Briker A., Kaestner K.H. (2017). High-fidelity Glucagon-CreER mouse line generated by CRISPR-Cas9 assisted gene targeting. Mol. Metab..

[24] Soriano P. (1999). Generalized lacZ expression with the ROSA26 Cre reporter strain. Nat. Genet..

[25] Collombat P., Mansouri A., Hecksher-Sorensen J., Serup P., Krull J., Gradwohl G., Gruss P. (2003). Opposing actions of Arx and Pax4 in endocrine pancreas development. Genes Dev..

[26] O’Rourke K.P., Dow L.E., Lowe S.W. (2016). Immunofluorescent Staining of Mouse Intestinal Stem Cells. Bio Protoc..

[27] Sato T., Vries R.G., Snippert H.J., van de Wetering M., Barker N., Stange D.E., van Es J.H., Abo A., Kujala P., Peters P.J. (2009). Single Lgr5 stem cells build crypt-villus structures in vitro without a mesenchymal niche. Nature.

[28] Eissele R., Goke R., Willemer S., Harthus H.P., Vermeer H., Arnold R., Goke B. (1992). Glucagon-like peptide-1 cells in the gastrointestinal tract and pancreas of rat, pig and man. Eur. J. Clin. Investig..

[29] Moolenbeek C., Ruitenberg E.J. (1981). The “Swiss roll”: A simple technique for histological studies of the rodent intestine. Lab. Anim..

[30] Habib A.M., Richards P., Cairns L.S., Rogers G.J., Bannon C.A., Parker H.E., Morley T.C., Yeo G.S., Reimann F., Gribble F.M. (2012). Overlap of endocrine hormone expression in the mouse intestine revealed by transcriptional profiling and flow cytometry. Endocrinology.

[31] Grodsky G.M., Bennett L.L. (1966). Cation requirements for insulin secretion in the isolated perfused pancreas. Diabetes.

[32] Belz M., Willenborg M., Gorgler N., Hamada A., Schumacher K., Rustenbeck I. (2014). Insulinotropic effect of high potassium concentration beyond plasma membrane depolarization. Am. J. Physiol. Endocrinol. Metab..

[33] Mellitzer G., Beucher A., Lobstein V., Michel P., Robine S., Kedinger M., Gradwohl G. (2010). Loss of enteroendocrine cells in mice alters lipid absorption and glucose homeostasis and impairs postnatal survival. J. Clin. Investig..

[34] Thompson E.M., Price Y.E., Wright N.A. (1990). Kinetics of enteroendocrine cells with implications for their origin: A study of the cholecystokinin and gastrin subpopulations combining tritiated thymidine labelling with immunocytochemistry in the mouse. Gut.

[35] Cheng H., Leblond C.P. (1974). Origin, differentiation and renewal of the four main epithelial cell types in the mouse small intestine. V. Unitarian Theory of the origin of the four epithelial cell types. Am. J. Anat..

[36] Garrido-Utrilla A., Ayachi C., Friano M.E., Atlija J., Balaji S., Napolitano T., Silvano S., Druelle N., Collombat P. (2022). Conversion of Gastrointestinal Somatostatin-Expressing D Cells Into Insulin-Producing Beta-Like Cells Upon Pax4 Misexpression. Front. Endocrinol..

[37] Gao N., White P., Kaestner K.H. (2009). Establishment of intestinal identity and epithelial-mesenchymal signaling by Cdx2. Dev. Cell.

[38] Bonis V., Rossell C., Gehart H. (2021). The Intestinal Epithelium—Fluid Fate and Rigid Structure From Crypt Bottom to Villus Tip. Front. Cell Dev. Biol..

[39] Pederson R.A., Satkunarajah M., McIntosh C.H., Scrocchi L.A., Flamez D., Schuit F., Drucker D.J., Wheeler M.B. (1998). Enhanced glucose-dependent insulinotropic polypeptide secretion and insulinotropic action in glucagon-like peptide 1 receptor -/- mice. Diabetes.

[40] Song Y., Koehler J.A., Baggio L.L., Powers A.C., Sandoval D.A., Drucker D.J. (2019). Gut-Proglucagon-Derived Peptides Are Essential for Regulating Glucose Homeostasis in Mice. Cell Metab..

[41] Capozzi M.E., Svendsen B., Encisco S.E., Lewandowski S.L., Martin M.D., Lin H., Jaffe J.L., Coch R.W., Haldeman J.M., MacDonald P.E. (2019). beta Cell tone is defined by proglucagon peptides through cAMP signaling. JCI Insight.

[42] Svendsen B., Larsen O., Gabe M.B.N., Christiansen C.B., Rosenkilde M.M., Drucker D.J., Holst J.J. (2018). Insulin Secretion Depends on Intra-islet Glucagon Signaling. Cell Rep..

[43] May C.L., Kaestner K.H. (2010). Gut endocrine cell development. Mol. Cell. Endocrinol..

[44] Barthel E.R., Speer A.L., Levin D.E., Sala F.G., Hou X., Torashima Y., Wigfall C.M., Grikscheit T.C. (2012). Tissue engineering of the intestine in a murine model. J. Vis. Exp..

[45] Liu Y., Wang Y., Chakroff J., Johnson J., Farrell A., Besner G.E. (2019). Production of Tissue-Engineered Small Intestine in Rats with Different Ages of Cell Donors. Tissue Eng. Part A.

[46] Kitamura Y.I., Kitamura T., Kruse J.P., Raum J.C., Stein R., Gu W., Accili D. (2005). FoxO1 protects against pancreatic beta cell failure through NeuroD and MafA induction. Cell Metab..

[47] Talchai C., Xuan S., Lin H.V., Sussel L., Accili D. (2012). Pancreatic beta cell dedifferentiation as a mechanism of diabetic beta cell failure. Cell.

[48] Link W., Fernandez-Marcos P.J. (2017). FOXO transcription factors at the interface of metabolism and cancer. Int. J. Cancer.

[49] Farhan M., Silva M., Xingan X., Huang Y., Zheng W. (2020). Role of FOXO Transcription Factors in Cancer Metabolism and Angiogenesis. Cells.

