# Supplementary material for: Harnessing Gut Endocrine Cell Plasticity to Restore Insulin Production
Source: Cells. 2026 Mar 19;15(6):544. doi: 10.3390/cells15060544 (PMC13026060; doi:10.3390/cells15060544)
Supplement: Supplementary file 1 [file cells-15-00544-s001.zip › Ayachi et al., 2026 - Supplemental Figure Legends - v2.pdf]

1. Supplemental Figure Legends

Figure S1. *Pax4* expression is significantly increased throughout the GI tract of Gcg-Cre^{ERT2}::Pax4-OE animals. (**A**) *Pax4* mRNA levels were assessed by RT-qPCR. Relative mRNA expression levels were normalized to GAPDH and expressed as fold-change relative to the fundus of control animals (n = 3–5). Statistical significance was determined using an unpaired t-test with Welch's correction (**$p < 0.01$, ***$p < 0.001$). Data are presented as mean ± SEM. Abbreviations: GI, gastrointestinal.

Figure S2. *Pax4* misexpression did not significantly affect the expression of other gut hormones in the colon. (**A**) *Sst*, *Pyy*, *Gastrin*, and *Gip* mRNA levels were assessed by RT-qPCR. Relative mRNA expression levels were normalized to GAPDH and expressed as fold-change relative to control animals (n = 5). Statistical significance was determined using an unpaired t-test with Welch's correction. Data are presented as mean ± SEM. Abbreviations: GIP, glucose-dependent insulinotropic polypeptide; PYY, peptide YY; SST, somatostatin.

Figure S3. *Pax4* misexpression in L-cells induces ectopic insulin$^+$ cell formation throughout the GI tract. (**A–F**) Immunohistochemical analysis of GI paraffin sections from TAM-treated Gcg-Cre^{ERT2}::Pax4-OE animals. Representative images show insulin (red). Nuclei were counterstained with DAPI (blue). Scale bar: 20 μm. Abbreviations: GI, gastrointestinal; INS, insulin; TAM, tamoxifen.

Figure S4. Lineage tracing analysis reveals the conversion of L-cells into insulin$^+$ cells upon Pax4 ectopic expression. (**A–B**) Immunohistochemical analysis of colonic paraffin sections from TAM-treated Gcg-Cre^{ERT2}::Pax4-OE animals and age- and sex-matched controls. Representative images show co-immunodetection of glucagon (green) and insulin (red), with yellow indicating colocalization. Nuclei were counterstained with DAPI (blue). Scale bar: 20 μm; (**C–D**) Co-immunohistochemical analysis of β-galactosidase (green) and insulin (red) on colonic paraffin sections from TAM-treated Gcg-CreERT2::Rosa26-β-gal control and Gcg-CreERT2::Pax4-OE mice, with yellow indicating colocalization. Nuclei were counterstained with DAPI (blue). Scale bar: 20 μm; (**E**) Quantification of the percentage of β-galactosidase$^+$ cells co-expressing insulin in the colon. A total of 100 β-galactosidase$^+$ cells were counted per mouse (n = 5). Data are presented as mean ± SEM. Abbreviations: β-GAL, β-galactosidase; GCG, glucagon; INS, insulin; TAM, tamoxifen.

Figure S5. Impaired GLP-1 secretion during oGTT in *Pax4*-misexpressing animals. (**A**) Plasma GLP-1 levels were measured at baseline and 10 and 15 min following oral glucose administration (2 g/kg) in control and TAM-treated Gcg-Cre^{ERT2}::Pax4-OE mice (n = 6–7). Statistical significance was determined using an unpaired t-test with Welch's correction (*$p < 0.05$). Data are presented as mean ± SEM. Abbreviations: oGTT, oral glucose tolerance test; GLP-1, glucagon-like peptide 1; TAM, tamoxifen.

Figure S6. Conversion of L-cells into β-like cells in *Pax4*-misexpressing colonoids. (**A**) Co-immunofluorescent detection of glucagon (green) and insulin (red) in Gcg-Cre^{ERT2}::Pax4-OE colonoids treated *in vitro* for 72 h with 10 μM 4-OHT, with yellow indicating colocalization. Nuclei were counterstained with DAPI (blue). Scale bar: 25 μm; (**B**) Co-immunohistochemical analysis of β-galactosidase (green) and insulin (red) in transgenic colonoids, with yellow indicating colocalization. Nuclei were counterstained with DAPI (blue). Scale bar: 25 μm; (**C–D**) Co-immunohistochemical analysis of insulin (red) together with PC1/3 (green), or C-peptide (green) in 4-OHT-treated colonoids, with yellow indicating colocalization. Nuclei were counterstained with DAPI (blue). Scale bar, 20 μm; (**E**) β-cell-associated markers mRNA levels were assessed by RT-qPCR. Relative mRNA

 https://doi.org/10.3390/xxxxx

expression levels were normalized to GAPDH and expressed as fold-change relative to control colonoids (n = 4–6). Statistical significance was determined using an unpaired t-test with Welch's correction (*$p < 0.05$, **$p < 0.01$, ***$p < 0.001$, ****$p < 0.0001$). Data are presented as mean ± SEM. Abbreviations: 4-OHT, 4-hydroxytamoxifen; β-GAL, β-galactosidase; C-PEP, C-peptide; GCG, glucagon; INS, insulin.

Figure S7. Pulse–chase analysis of glucose tolerance following tamoxifen washout and re-administration**.** (**A**) ipGTT (2 g/kg) and corresponding AUC analysis performed after a 7-day TAM washout in control and previously TAM-induced Gcg-Cre^{ERT2}::Pax4-OE animals (n = 6–7); (**B**) ipGTT (2 g/kg) and corresponding AUC analysis performed following 5 days of TAM re-administration in control and Gcg-Cre^{ERT2}::Pax4-OE animals (n = 6–7). Statistical significance for GTT curves and AUC analyses was determined using a two-way ANOVA with Bonferroni's correction and an unpaired t-test with Welch's correction, respectively (*$p < 0.05$, **$p < 0.01$). Data are presented as mean ± SEM. Abbreviations: AUC, area under the curve; ipGTT, intraperitoneal glucose tolerance test; TAM, tamoxifen.

Figure S8. *Pax4* ectopic expression in α-cells does not induce insulin$^+$ cell formation in pancreatic islets. (**A**) Immunohistochemical analysis of pancreatic paraffin sections from TAM-treated Gcg-Cre^{ERT2}::Pax4-OE mice. Representative image show β-galactosidase (green) and insulin (red). Nuclei were counterstained with DAPI (blue). Scale bar: 50 μm; (**B**) *Pax4* and *insulin* mRNA levels were assessed by RT-qPCR. Relative mRNA expression levels were normalized to GAPDH and expressed as fold-change relative to control isolated islets (n = 3). Statistical significance was determined using an unpaired t-test with Welch's correction (**$p < 0.01$). Data are presented as mean ± SEM. Abbreviations: β-GAL, β-galactosidase; INS, insulin; TAM, tamoxifen.

https://doi.org/10.3390/xxxxx
