# Supplementary figures and images for: Harnessing Gut Endocrine Cell Plasticity to Restore Insulin Production
Source: Cells. 2026 Mar 19;15(6):544. doi: 10.3390/cells15060544 (PMC13026060; doi:10.3390/cells15060544)

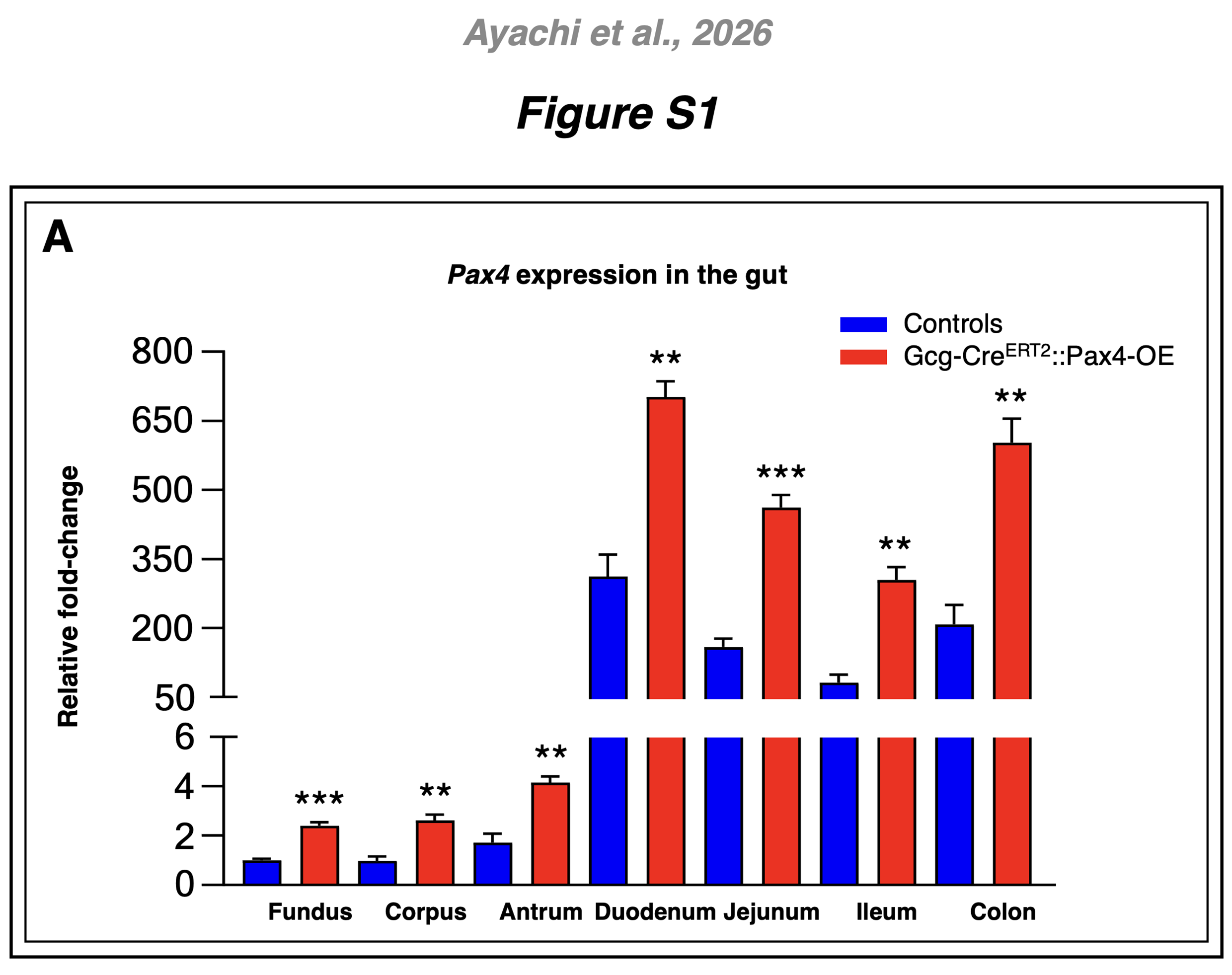

Supplement: Supplementary file 1 [file cells-15-00544-s001.zip › Ayachi et al. 2026 - Figure S1.tif]

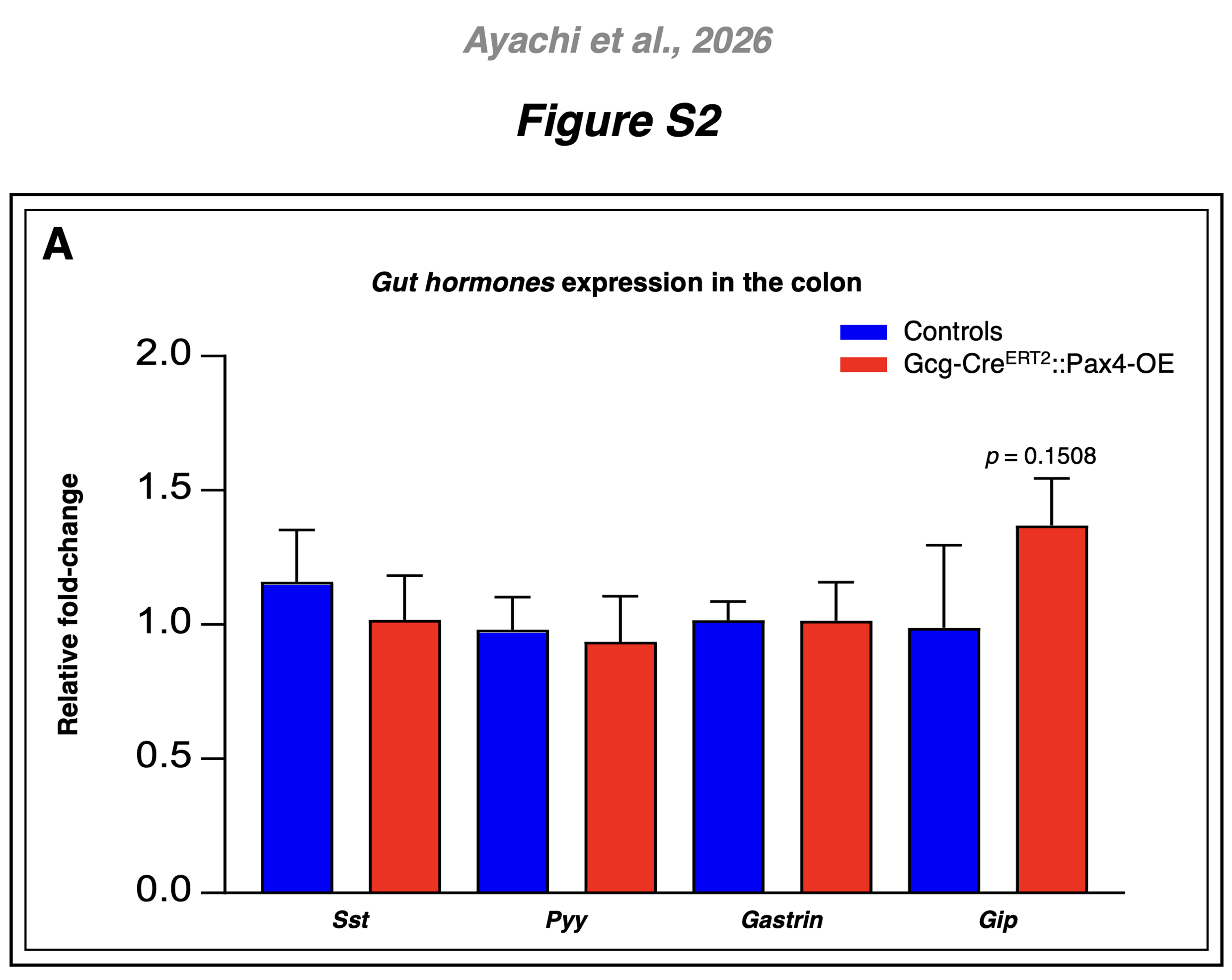

Supplement: Supplementary file 1 [file cells-15-00544-s001.zip › Ayachi et al. 2026 - Figure S2.tif]

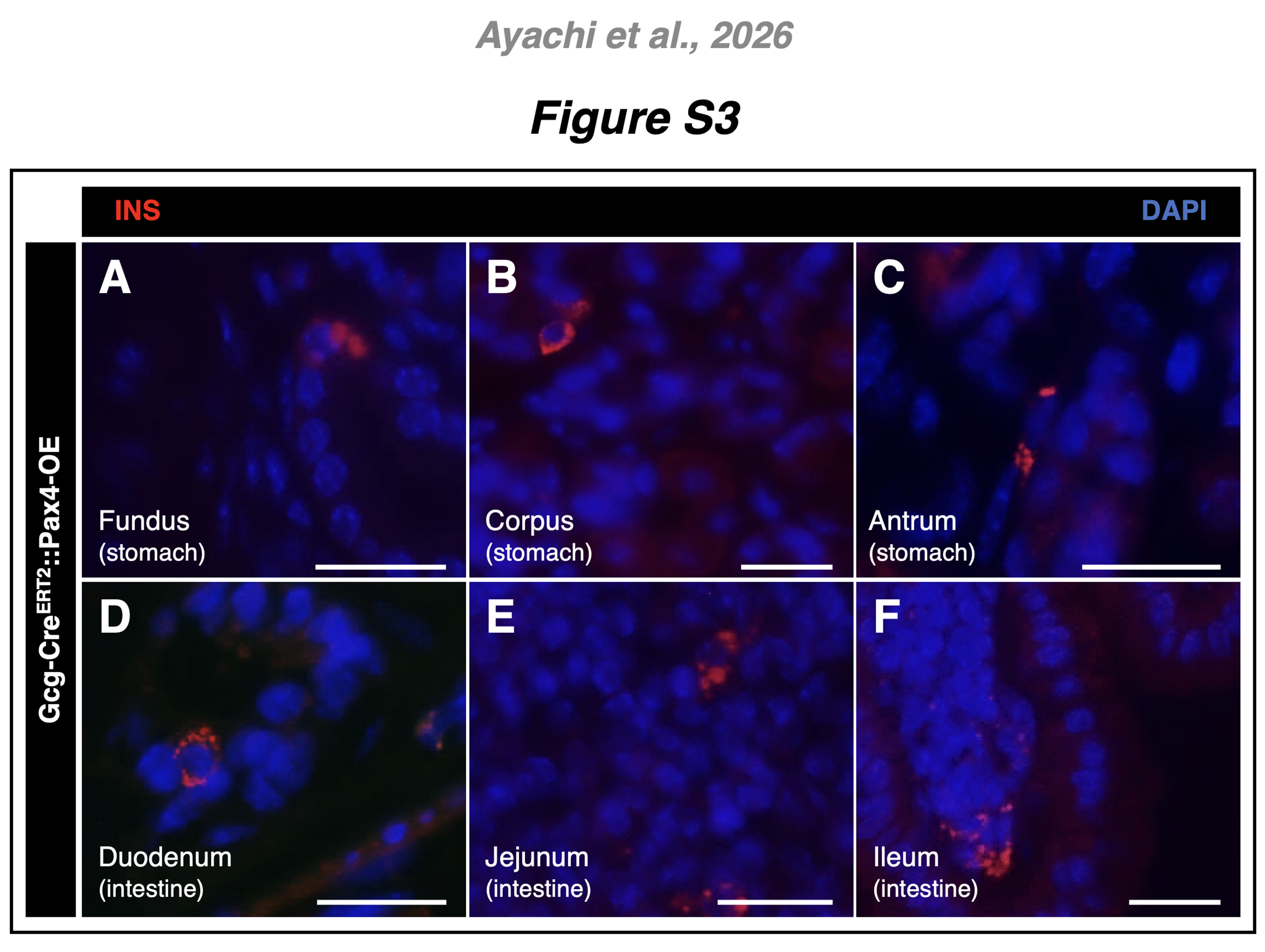

Supplement: Supplementary file 1 [file cells-15-00544-s001.zip › Ayachi et al. 2026 - Figure S3.tif]

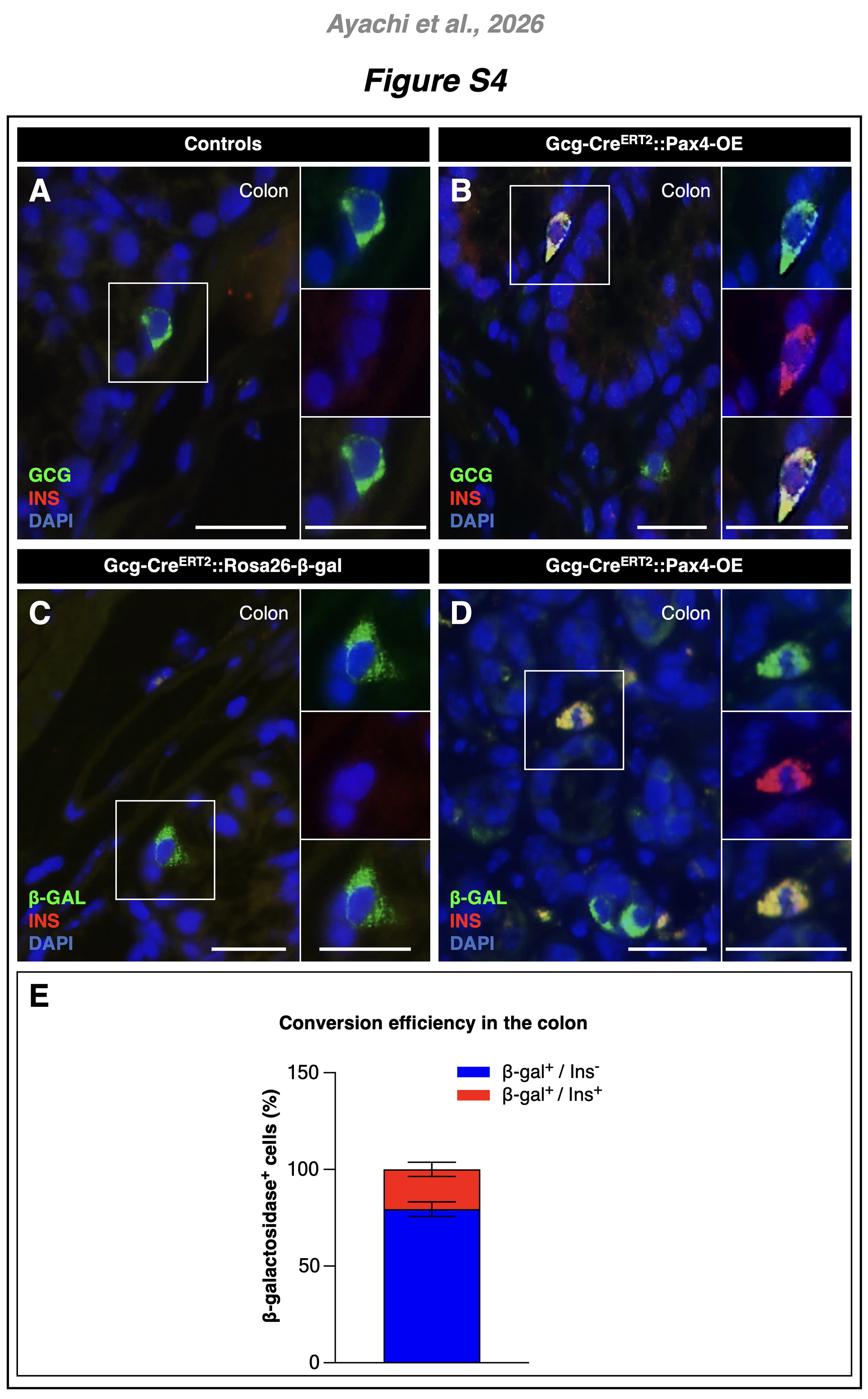

Supplement: Supplementary file 1 [file cells-15-00544-s001.zip › Ayachi et al. 2026 - Figure S4.tif]

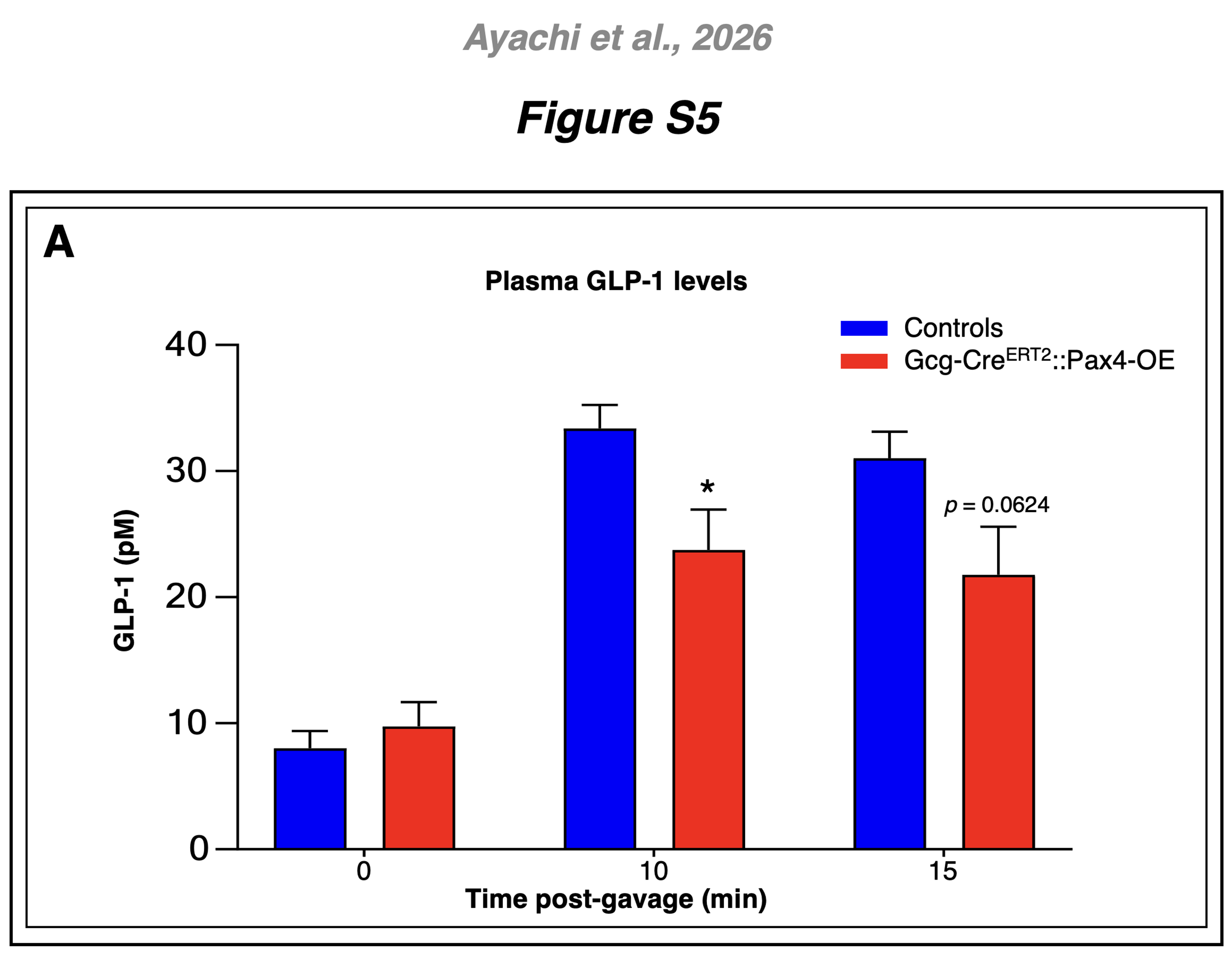

Supplement: Supplementary file 1 [file cells-15-00544-s001.zip › Ayachi et al. 2026 - Figure S5.tif]

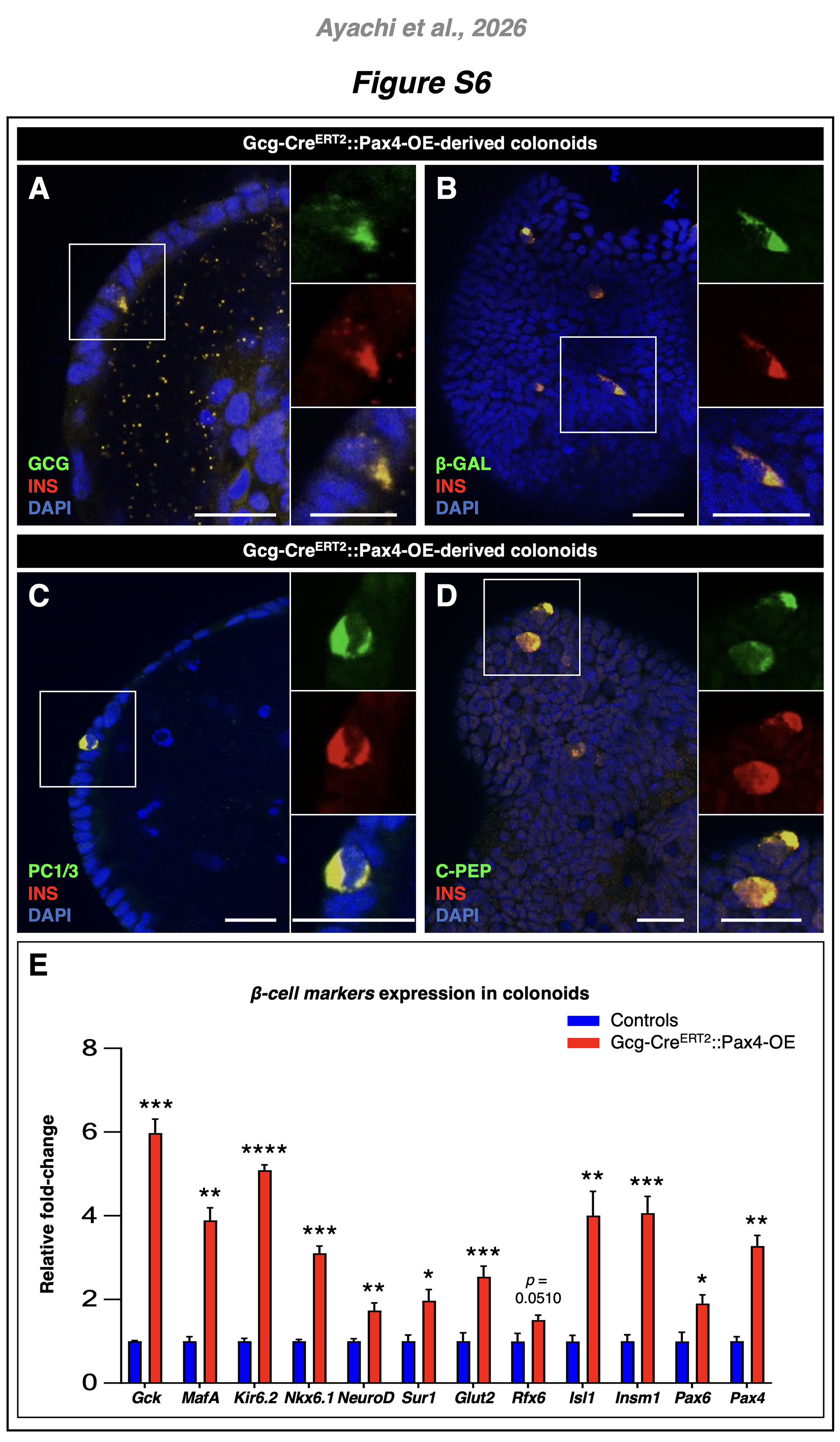

Supplement: Supplementary file 1 [file cells-15-00544-s001.zip › Ayachi et al. 2026 - Figure S6.tif]

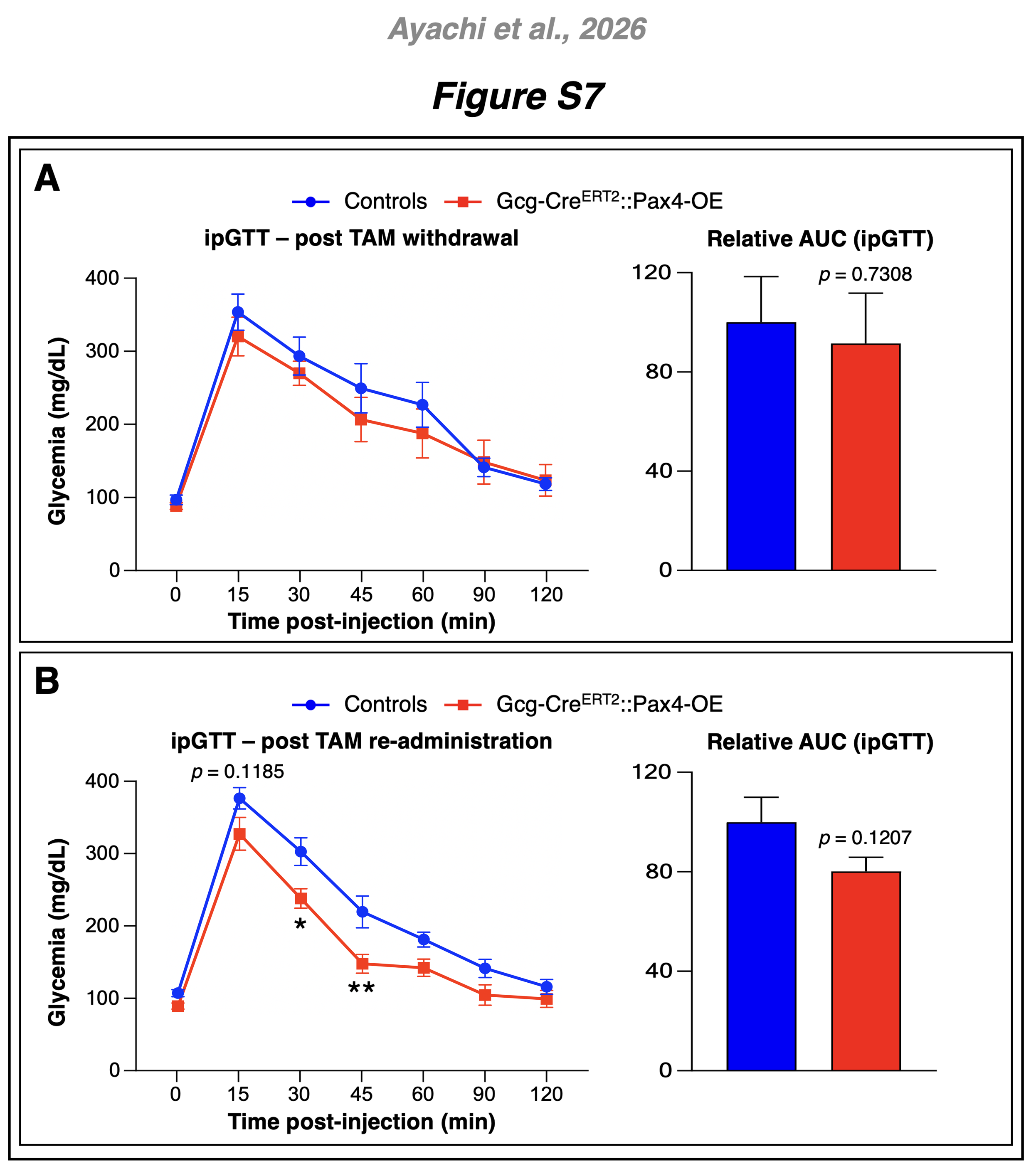

Supplement: Supplementary file 1 [file cells-15-00544-s001.zip › Ayachi et al. 2026 - Figure S7.tif]

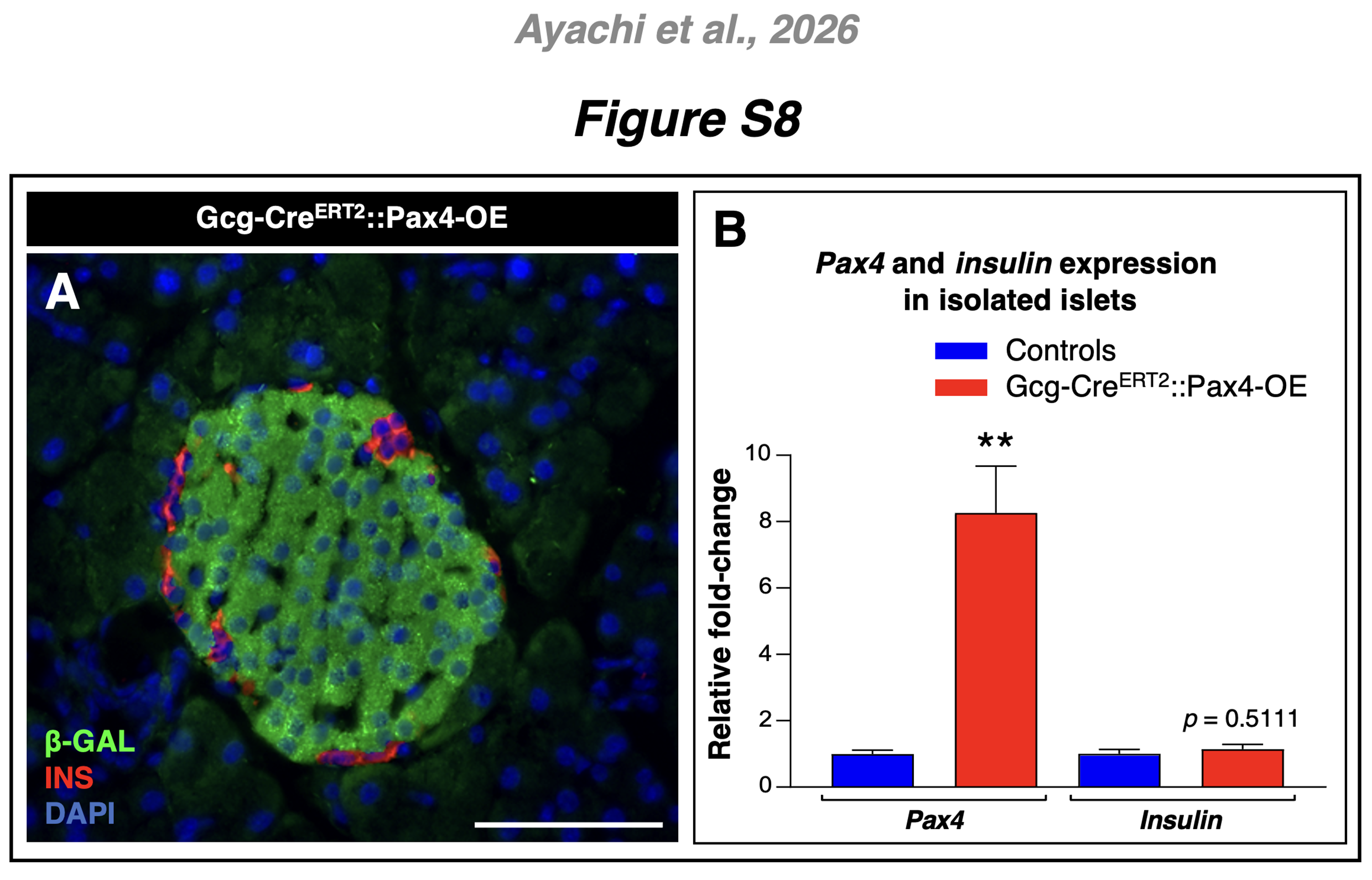

Supplement: Supplementary file 1 [file cells-15-00544-s001.zip › Ayachi et al. 2026 - Figure S8.tif]
